# NDUFB7 mutations cause brain neuronal defects, lactic acidosis, and mitochondrial dysfunction in humans and zebrafish

**DOI:** 10.1038/s41420-025-02369-0

**Published:** 2025-03-01

**Authors:** Yen-Lin Chen, Brian Hon-Yin Chung, Masakazu Mimaki, Shumpei Uchino, Yin-Hsiu Chien, Christopher Chun-Yun Mak, Steven Shinn-Forng Peng, Wei-Chen Wang, Yu-Li Lin, Wuh-Liang Hwu, Shyh-Jye Lee, Ni-Chung Lee

**Affiliations:** 1 Department of Life Science, National Taiwan University, Taipei, 10617 Taiwan; 2 Department of Pediatrics and Adolescent Medicine, LKS Faculty of Medicine, The University of Hong Kong, Hong Kong, 999077 China; 3 Department of Pediatrics, Teikyo University School of Medicine, Tokyo, 173-8605 Japan; 4 Department of Pediatrics, The University of Tokyo, Tokyo, 113-8655 Japan; 5 Department of Medical Genetics, National Taiwan University Hospital, Taipei, 10041 Taiwan; 6 Department of Pediatrics, National Taiwan University Hospital and National Taiwan University College of Medicine, Taipei, 10041 Taiwan; 7 Department of Radiology, National Taiwan University Hospital and National Taiwan University College of Medicine, Taipei, 10002 Taiwan; 8 Department of Medical Research, National Taiwan University Hospital, Taipei, 10041 Taiwan; 9 Center for Biotechnology, National Taiwan University, Taipei, 10617 Taiwan; 10 Research Center for Developmental Biology and Regenerative Medicine, National Taiwan University, Taipei, 10617 Taiwan

**Keywords:** Neurodevelopmental disorders, Metabolic disorders

## Abstract

Complex I of the mitochondrial electron transfer chain is one of the largest membrane protein assemblies ever discovered. A patient carrying a homozygous NDUFB7 intronic mutation died within two months after birth due to cardiorespiratory defects, preventing further study. Here, we report another patient with compound heterozygous mutations in *NDUFB7* who suffers from pons abnormality, lactic acidosis, prematurity, prenatal and postnatal growth deficiency, incomplete closure of the abdominal wall (ventral hernia), and a poorly functioning gastrointestinal tract (pseudo-obstruction). We demonstrated that the patient’s skin fibroblasts are deficient in Complex I assembly and reduced supercomplex formation. This report further broadens the spectrum of mitochondrial disorders. The patient has had several surgeries. After receiving treatment with Coenzyme Q10 and vitamin B complex, she has remained stable up to this point. To further explore the functionality of NDUFB7 in vivo, we knocked down Ndufb7 in zebrafish embryos. This resulted in brain ventricle and neuronal defects, elevated lactic acid levels, and reduced oxygen consumption, indicating defective mitochondrial respiration. These phenotypes can be specifically rescued by ectopic expression of *ndufb7*. More importantly, Mitoquinone mesylate (MitoQ), a common remedy for mitochondrial disorders, can ameliorate these conditions. These results suggest a role for NDUFB7 in mitochondrial activity and the suitability of the zebrafish model for further drug screening and the development of therapeutic strategies for this rare disease.

## Introduction

Mitochondrial disorders are a clinically and genetically heterogeneous group of diseases caused by mutations in mitochondrial or nuclear gene-encoded mitochondrial respiratory chain genes. Among the five enzyme complexes of the respiratory chain, Complex I (NADH:ubiquinone oxidoreductase) is the first on the chain and is one of the biggest and most fragile membrane protein assemblies ever discovered [1]. The eukaryotic Complex I is located in the inner membrane of the mitochondrion, and the mammalian complex I is composed of 45 different subunits (14 conserved core subunits from bacteria to humans, 31 accessory subunits) [2]. The core subunits and 9 cofactors (1 flavin mononucleotide and 8 iron-sulfur clusters) are essential for the catalytic function of this complex [2–4]. The 31 accessory subunits encoded in the nuclear genome are important for the complex stability [5–7]. These accessory subunits are essential for functionally active complex I [6, 7]. Mutations in all 14 Complex I core subunits and 10 of the 11 assembly protein genes are associated with mitochondrial disorders [8]. Mutation of subunits will lead to inherited metabolic and neuromuscular disorders and cause diseases such as cardiac infarction and Parkinson’s disease [9]. Mutations in 22 of the 31 accessory protein genes have been associated with mitochondrial disorders causing lactic acidosis, Leigh/Leigh-like syndrome, cardiomyopathy, cardiac hypertrophy, and cerebellar atrophy [8, 10–25]. The biological functions of other accessory subunits are still not defined.

Correia et al. recently reported a patient carrying an intronic mutation in the NDUFB7 gene [21]. The mutation was homozygous and caused severe congenital lactic acidosis and hypertrophic cardiomyopathy. The NDUFB7 is one of the accessory proteins (NDUFB2, NDUFB3, NDUFB7, NDUFB8, and NDUFB9) together with the central subunit ND5 to assemble a PD‐b, the last module of mitochondrial complex 1 [26**–**31]. Biochemical analysis of the patient-derived fibroblasts revealed a decrease in NDUFB7, which correlated with a reduction in NDUFB8 (part of the PD-b module) as well as proteins from the Q module (NDUFS3) but not with proteins from the N module (NDUFV1). This indicates an essential role of NDUFB7 in Complex I assembly, as previously suggested. More critically, the loss of NDUFB7 is likely pathogenic. By mining the NDUFB7 variants on the ClinVar Miner (https://clinvarminer.genetics.utah.edu/), we found one pathogenic *NDUFB7* (c.113-10C>G), which was reported by Correia et al. as described [21]. Only four additional unspecified NDUFB7 variants were submitted: c.13C>G (p.Leu5Val), c.55C>T (p.Pro19Ser), c.292C>G (p.Arg98Gly), and c.295A>C (p.Met99Leu). It suggests the rarity of NDUFB7 variants and the need for animal models for disease mechanistic studies.

Here, we present a unique case of a girl with compound heterozygous mutations in the NDUFB7 gene. Unlike the previously described patient with a homozygous NDUFB7 intronic mutation, this patient still survives, providing a rare opportunity for long-term observation and the potential development of therapeutic approaches for human mitochondrial disorders. Furthermore, to examine the biological functions of Ndufb7, we utilized zebrafish as a model to analyze the phenotypes by knocking down Ndufb7.

## Results

### Clinical report

A girl patient was born to healthy, non-consanguineous parents with premature delivery (gestational age 34 weeks) and low birth weight (1150 g, <3rd percentile). After birth, she was noted to have feeding difficulties, ventral hernia, hypotonia, esotropia, poor growth, and developmental delay. Her karyotype was normal. Orogastric tube feeding was required from birth because of oral dysphagia, gastroesophageal reflux, and delayed gastric emptying. Surgery was performed at 8 months of age for gastrostomy and herniorrhaphy, and a 1-cm peritoneal defect was found during the surgery. A muscle biopsy at 8 months of age revealed neurogenic myopathy. Another surgery was performed at 5 years old for the closure of gastrostomy. At the age of 12.5 years, her body height (126.8 cm, <3rd percentile) and weight (22.4 kg, <3rd percentile) were still very low. She also had hypogonadotropic hypogonadism, mild intellectual disability, and attention-deficit/hyperactivity disorder.

**Fig. 1:**
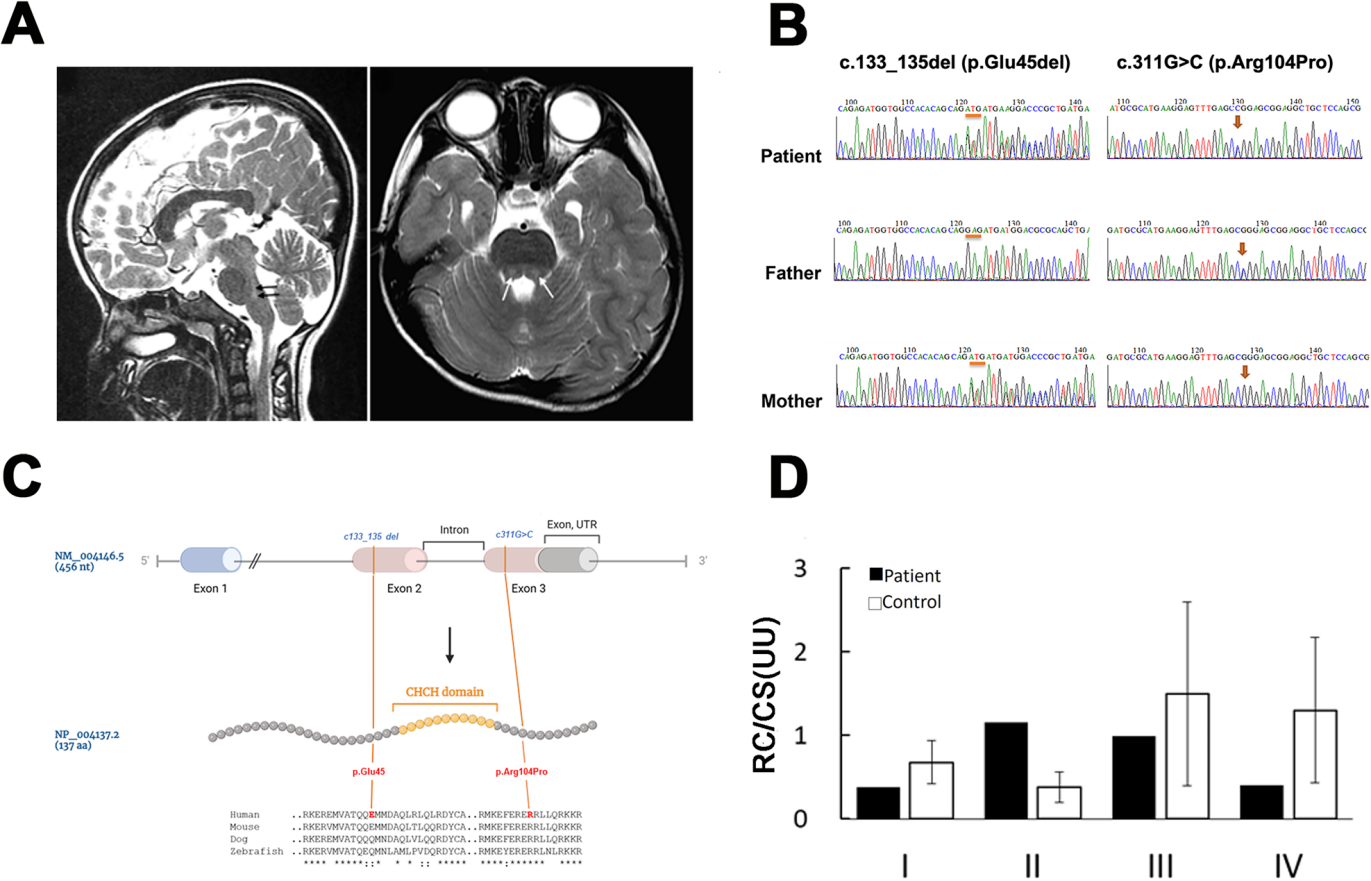
NDUFB7 is the disease causative gene. **A** The patient’s brain MRI T2-weighted sagittal (left) or axial image obtained at 4 years old reveals high-intensity pons lesions (gray and pointed by arrows). **B** Partial Sanger sequencing chromatographs of the patient and parents. The left panel shows the presence of a deletion (denoted by orange bars) in the patient and her mother, while the right panel shows a missense mutation (denoted by orange arrows) found in the patient and her father. **C** The upper panel shows the gene structure of the human NDUFB7 gene (NM_004146.5, 456 nucleotides (nt)), which has three exons and two introns (the intron between Exon 2 and 3 is indicated). The orange lines indicate two mutations in Exon 2 and 3 of the patient’s NDUFB7 gene. The NDUFB gene is translated to 137 amino acids (aa, NP_004137.2, a cartoon in the middle panel). A large portion of Exon 2 and 3 encodes a coiled-coil-helix-coiled-coil-helix (CHCH) domain marked in orange. The lower panel presents a sequence alignment of sequences comprising the mutations and the CHCH domains among human, mouse, dog, and zebrafish (“*” identical; “:” similar). **D** Respiratory chain (RC) activities in cultured fibroblasts from the patient expressed as a ratio (U/U) relative to citrate synthase (CS) activity. The patient’s cells exhibited higher Complex II activity but lower activities in other complexes.

Her baseline plasma lactate levels were 3–5 mM (normal < 2 mM) with a normal lactate/pyruvate molar ratio (17.96, normal 10–20). Three episodes of acute exacerbation of lactic acidosis (8–11 mM) occurred at ages 8 months, 2 years, and 4 years. During these episodes, the elevation of alanine transaminase (ALT; up to 114 U/L), aspartate transaminase (AST; up to 74 U/L), blood ketone (up to 3.5 mmol/L, normal < 0.2 mM), and plasma alanine (up to 1020 μM, normal 159–594 μM) levels were noted. Her creatine kinase and blood sugar levels were normal. Brain magnetic resonance imaging (MRI) obtained at age 4 exhibited T2-weighted high-intensity lesions at the pons (Fig. 1A) and mild dilatation of the lateral ventricles. Because of the suspicion of mitochondrial disease, a repeated muscle biopsy was performed at 5 years of age, but no ragged-red fibers were present.

### NDUFB7 is the disease causative gene

To search for the potential gene mutations of the patient, we performed whole-exome sequencing using blood samples collected from the patient at age 12. Interestingly, two compound heterozygous mutations (g.14677723_14677725del; g.14677048C>G) were identified in a mitochondrial accessory protein gene, *NDUFB7* (NM_004146.5). Both mutations were validated by Sanger sequencing. In addition, we found that the c.133_135 del and the c311G>C missense mutation are genetically inherited from her father and mother, respectively (Fig. 1B). As shown in Fig. 1C, the human *NDUFB7* has 456 nucleotides with 3 exons. It encodes a protein of 137 amino acids with a coiled-coil-helix-coiled-coil-helix (CHCH) domain [32]. The deletion (c.133_135del; p.Glu45del) is before the Exon 2, and the missense mutation (c.311G>C; p.Arg104Pro) is after the Exon 3. The changed amino acid residues are flanking the CHCH domain, an evolutionarily conserved region shown in a sequence alignment among humans, mice, dogs, and zebrafish (Fig. 1C). The missense mutation was predicted to be deleterious using SIFT (http://sift.jcvi.org/), PolyPhen-2 (http://genetics.bwh.harvard.edu/pph2/), and PROVEAN (https://provean.jcvi.org/).

We subjected the wildtype and two mutated NDUFB7 sequences to AlphaFold (https://alphafoldserver.com/) for structural prediction and comparison (Supplementary Fig. 1). Structural alignments were conducted and visualized with ChimeraX to assess their similarity. The sequence alignment score between the wild-type protein and the c133_135 del variant is 702.1, with a Root-Mean-Square Deviation (RMSD) of 0.543 Å (<2 Å is highly similar). Similarly, the sequence alignment score between the wild-type protein and the c311 G>C variant is 713.4, with an RMSD of 0.418 Å. These findings indicate that both variants maintain high structural similarity to the wild-type protein.

NDUFB7 is an integral accessory protein of mitochondrial Complex I. To investigate its role, we cultured fibroblasts from the patient and a healthy control to assess their respiratory chain activities. The patient’s cells exhibited higher Complex II activity but reduced activities in other complexes (Fig. 1D). These results suggest that the NDUFB7 gene is the causative factor underlying the patient’s disorder.

After the diagnosis, the patient received Ubidecarenone (coenzyme Q10) and vitamin B complex. She is now 20 years old, with short stature, delayed puberty, attention and hyperactivity, and pre-diabetic mellius status (fasting glucose 110–130 mg/dl, HbA1C 5.9–6.1%). A fluctuating lactate level (2–6 mM) was noted without acute decompensation.

### Mitochondrial complex formation is disrupted in fibroblasts derived from the patient carrying the NDUFB7 mutations

**Fig. 2:**
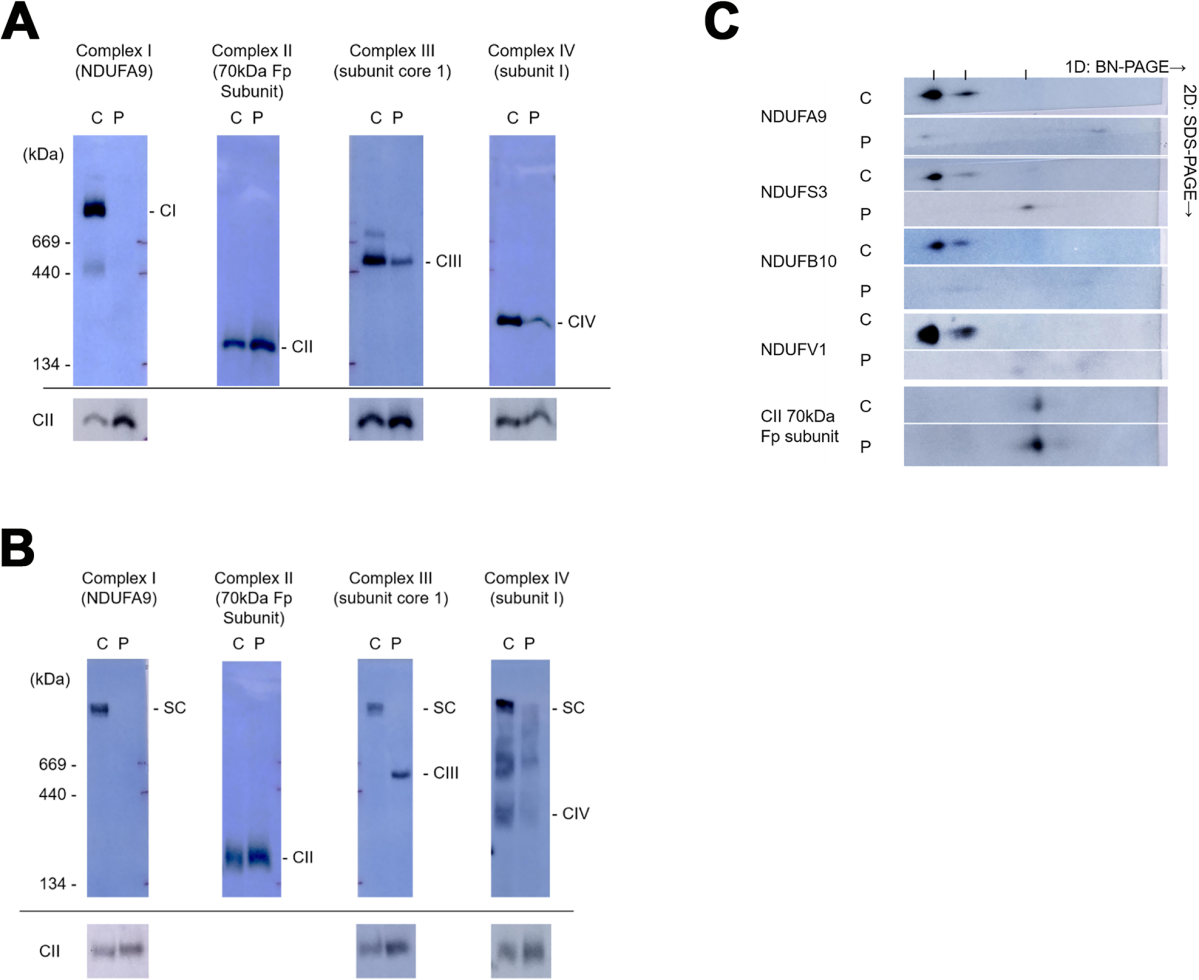
Mitochondrial complex formation is disrupted in fibroblasts derived from the patient carrying the NDUFB7 mutations. Using blue native polyacrylamide gel electrophoresis (BN-PAGE), mitochondrial extracts from control (C) and patient (P) skin fibroblasts were separated using Triton **A** X-100 or **B** digitonin. These extracts were probed for the indicated protein in parentheses to identify Complexes I–IV. Complex II served as a loading control, as shown at the bottom of each well, except in the Complex II lane. **C** The samples were further separated by 2D-SDS PAGE and probed for the indicated mitochondrial accessory proteins.

To further examine the effects of NDUFB7 mutations on the mitochondrial complexes, we performed Blue native PAGE (BN-PAGE) on mitochondrial extracts purified from the patient’s skin fibroblasts (Fig. 2). One-dimensional analysis revealed that Complex I is not visible in the patient’s sample using a strong detergent Triton X-100. Complexes III and IV amounts are also reduced (Fig. 2A). A mild detergent digitonin was used to examine super-complexes. It showed that super-complexes I, III, and IV are significantly reduced in the patient (Fig. 2B). Two-dimensional BN-PAGE further demonstrated a profound decrease in Complex I subunit levels (Fig. 2C). In the patient’s mitochondria, NDUFS3, which is part of the Q module of Complex I, was detected in the low-molecular-weight subcomplex. However, the patient’s fibroblasts had no signals for NDUFB10 (in the membranous P module) and NDUFV1 (in the N module).

### Knockdown of Ndufb7 disturbs the formation of the brain ventricle in zebrafish

**Fig. 3:**
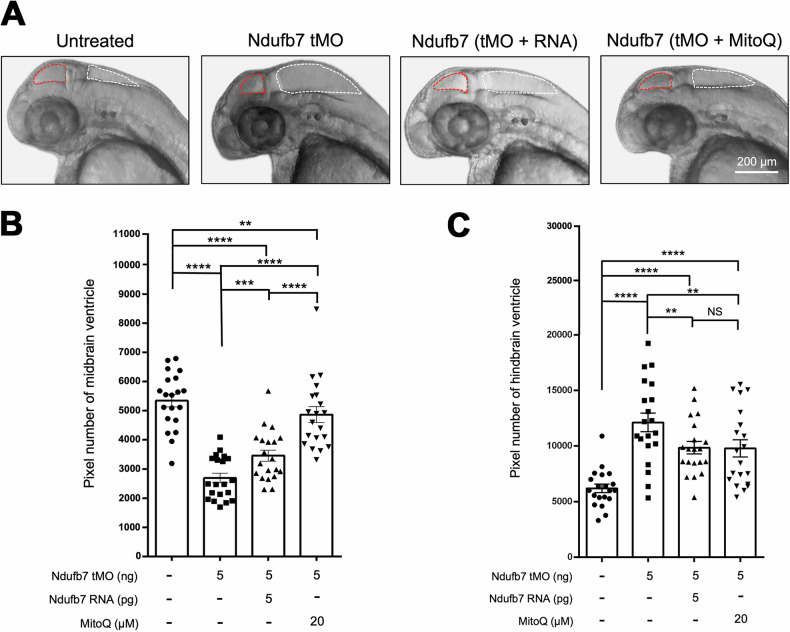
Knockdown of Ndufb7 changes the sizes of brain ventricles. We microinjected 1-cell stage zebrafish embryos without (untreated) or with indicated reagents and photographed them under a stereomicroscope at 48 hours post-fertilization. **A** Representative images for each treatment are presented. The ventricles are enclosed in red and white dotted lines for the midbrain and hindbrain, respectively. The area of the ventricle was measured in pixels using the Image J software. All data are shown in a scatter plot with mean ± standard error of the mean (SEM) for each treatment shown at the bottom of each column for **B** midbrain and **C** hindbrain, respectively, from three independent experiments. **p < 0.01; ***p < 0.001; ****p < 0.0001.

**Fig. 4:**
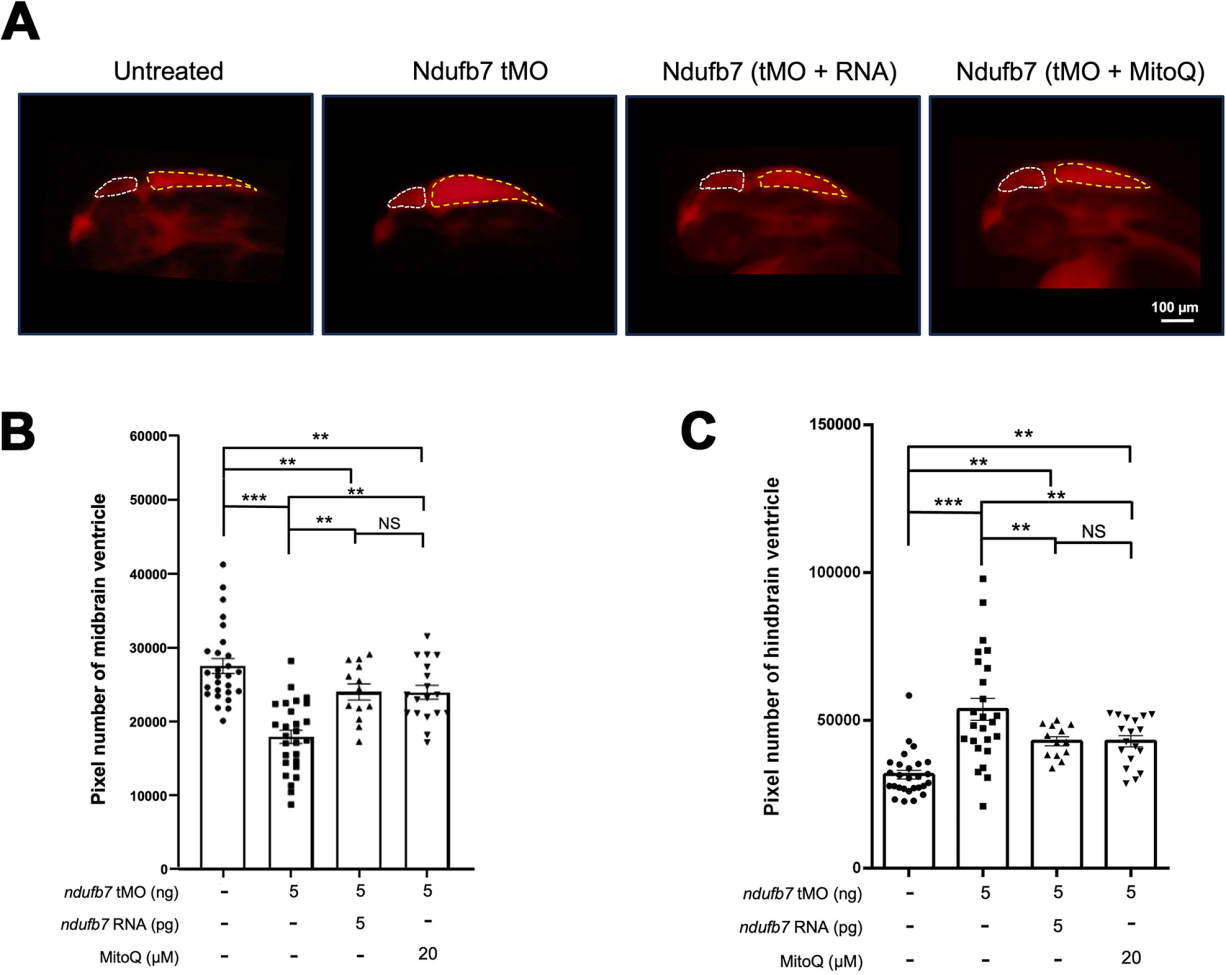
Knockdown of Ndufb7 changes the sizes of brain ventricles revealed by dextran rhodamine injection. **A** Zebrafish embryos were treated as described in Fig. 3, cultured until 48 hours post-fertilization, injected with dextran rhodamine into their brain ventricles, and photographed under a black field using a rhodamine cube. Representative images are presented in lateral view. **B**, **C** The midbrain (smaller chamber at the anterior) and hindbrain (larger chamber at the posterior) are enclosed by dotted lines. The area of the ventricle was measured in pixels, analyzed, and shown as described in Fig. 3 from three independent experiments. NS: not significant; **p < 0.01; ***p < 0.001.

We chose zebrafish to build a Ndufb7-deficiency animal model because the protein sequence alignment shows high homology among humans, dogs, mice, and zebrafish in the NDUFB7 genes covering the patient’s mutation sites (Fig. 1C). We microinjected a translational-blocking morpholino oligonucleotide (tMO) against the *ndufb7* at 5 ng per embryo into 1-cell stage zebrafish embryos, cultured to 48 hours post-fertilization (hpf). The Ndufb7 tMO-treated embryos (will be called morphants hereafter) show malformed brain ventricles (Fig. 3A). We measured their ventricle sizes in Image J, and found significantly reduced midbrain but enlarged hindbrain area in Ndufb7 morphants compared to the untreated controls (Fig. 3B, C, respectively). To validate whether the specific loss of Ndufb7 causes the phenotypes, we co-injected Ndufb7 mRNA (5 pg per embryo) with tMO and found a partial rescue of ventricle sizes. Furthermore, we found that the co-injection of 20 µM Mitoquinone mesylate (MitoQ), a mitochondria antioxidant, can significantly improve the rescue of midbrain size (Fig. 3B) and has similar rescue efficacy on the hindbrain (Fig. 3C). To validate the change in brain ventricle sizes in Ndufb7-morphants, we microinjected rhodamine dextran into brain ventricle of 48-hpf zebrafish embryos. We observed similar malformation of brain ventricles and their rescue by Ndufb7 mRNA and MitoQ compared to that in Fig.3 (Fig. 4). The alleviation by MitoQ suggests the loss of Ndufb7 may result in mitochondrial dysfunction and, thus, a reduction of aerobic respiration to disturb the brain development in zebrafish.

### Knockdown of Ndufb7 reduces the neuronal volume of zebrafish brain

**Fig. 5:**
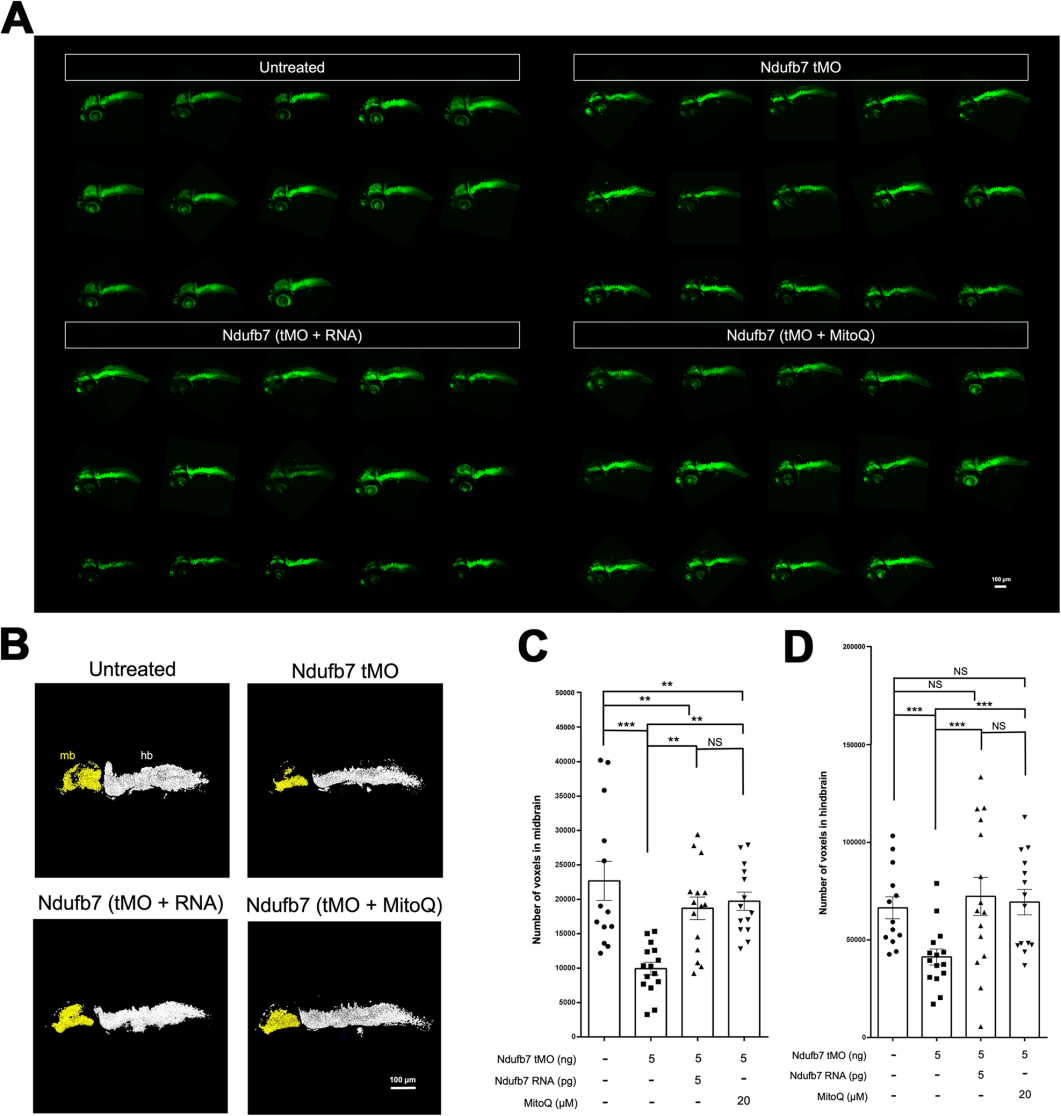
Knockdown of Ndufb7 reduces the neuronal volume of the brain. Transgenic Tg(*Huc:kaede*) zebrafish embryos were treated as described in Fig. 3, cultured until 48 hours post-fertilization, observed using a 10X objective, and photographed under a black field using a GFP cube under confocal microscopy. **A** All images are presented in lateral view. **B** The midbrain (mb) and hindbrain (hb) are pseudocolored in yellow and white, subjected to volume measurement using the Metamorph software, as shown in **C** midbrain and **D** hindbrain. The volume of each brain compartment was measured in voxels. Data from three independent experiments are presented, analyzed, and shown as described in Fig. 3. NS not significant; **p < 0.01; ***p < 0.001.

To examine whether brain neurons are affected, which may be induced by mitochondria dysfunction, we treated *Tg(Huc:kaede)* zebrafish embryos, which express green fluorescence in most neurons [33], with Ndufb7 tMO and Ndufb7 mRNA or MitoQ as described, cultured to 48 hours post-fertilization, subjected to confocal microscopy, and photographed under black field (Fig. 5A). We measured the volume of brain neurons emitting green fluorescence Kaede in the midbrain and hindbrain (Fig. 5B) in a voxel, volume pixel. We found that Ndufb7-morphants have significantly reduced volumes in the midbrain (pseudocolor in yellow) and hindbrain neurons (pseudocolor in gray) compared to untreated embryos. The volume reduction could also be rescued by the Ndufb7 mRNA and MitoQ (Fig. 5B–D).

### Increase in lactic acid in Ndufb7-morphants

**Fig. 6:**
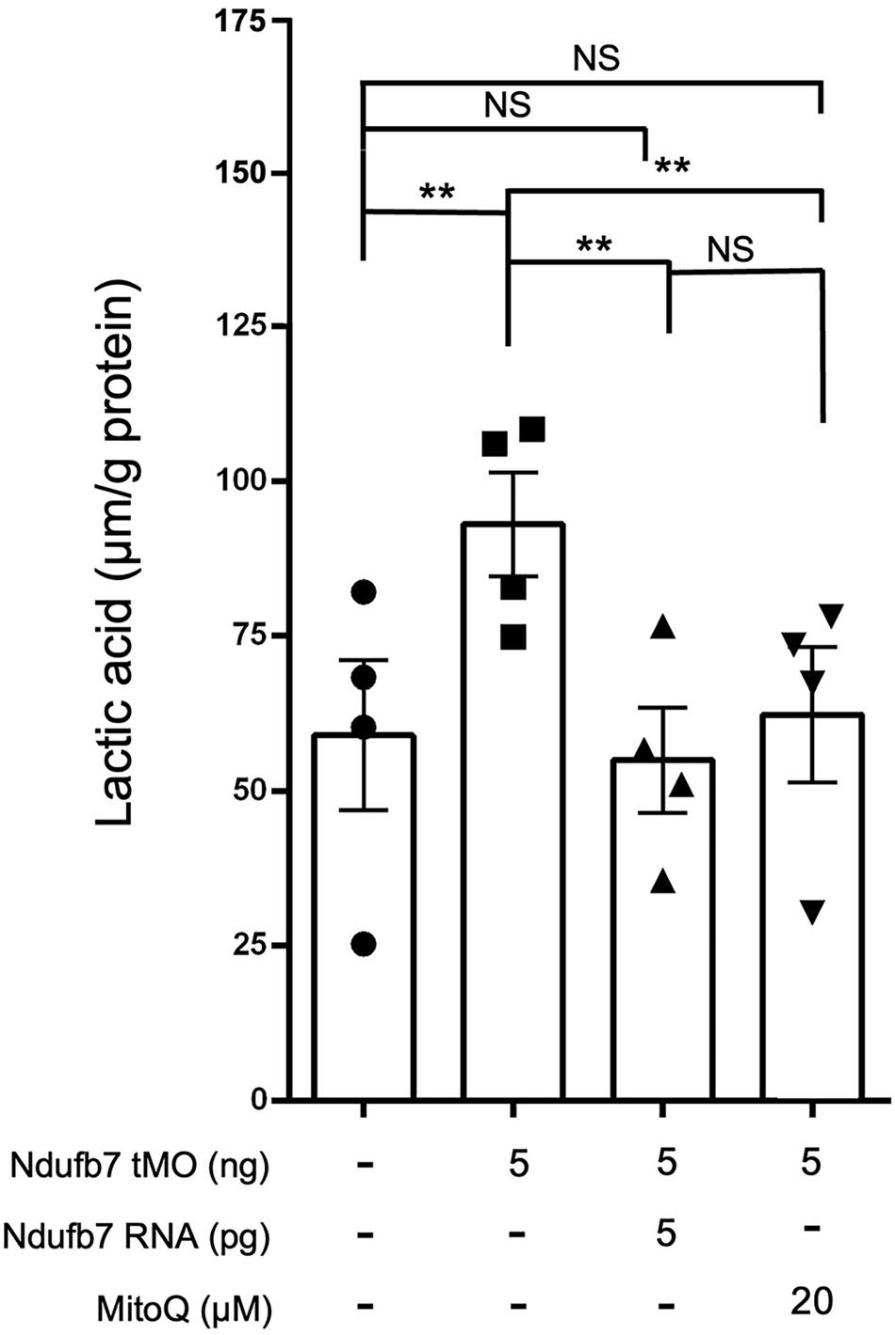
Knockdown of Ndufb7 increases the lactic acid in zebrafish embryos. We injected 1-cell stage zebrafish embryos without or with indicated reagents as described in Fig. 3, cultured to 24 h post fertilization, lyzed, and subjected to lactic acid measurement. Data from four independent experiments are presented, analyzed, and shown as described in Fig. 3. NS not significant; **p < 0.01.

The elevated blood lactate level in the patient suggests an increase in anaerobic respiration. To examine whether a similar phenotype exists in zebrafish, we measured the lactic acid contents of Ndufb7-morphants. The results showed that the lactic acid content is 59 and 93 µmol/g protein in the untreated control embryos and Ndufb7-morphants, respectively. Intriguingly, both Ndufb7 mRNA and MitoQ treatments could fully restore the lactic acid level (Fig. 6). These data indicate that the elevated lactic acid level is due to the loss of Ndufb7 which implies an increase in anaerobic respiration due to mitochondrial dysfunction.

### Metabolic defect in Ndufb7-morphants

**Fig. 7:**
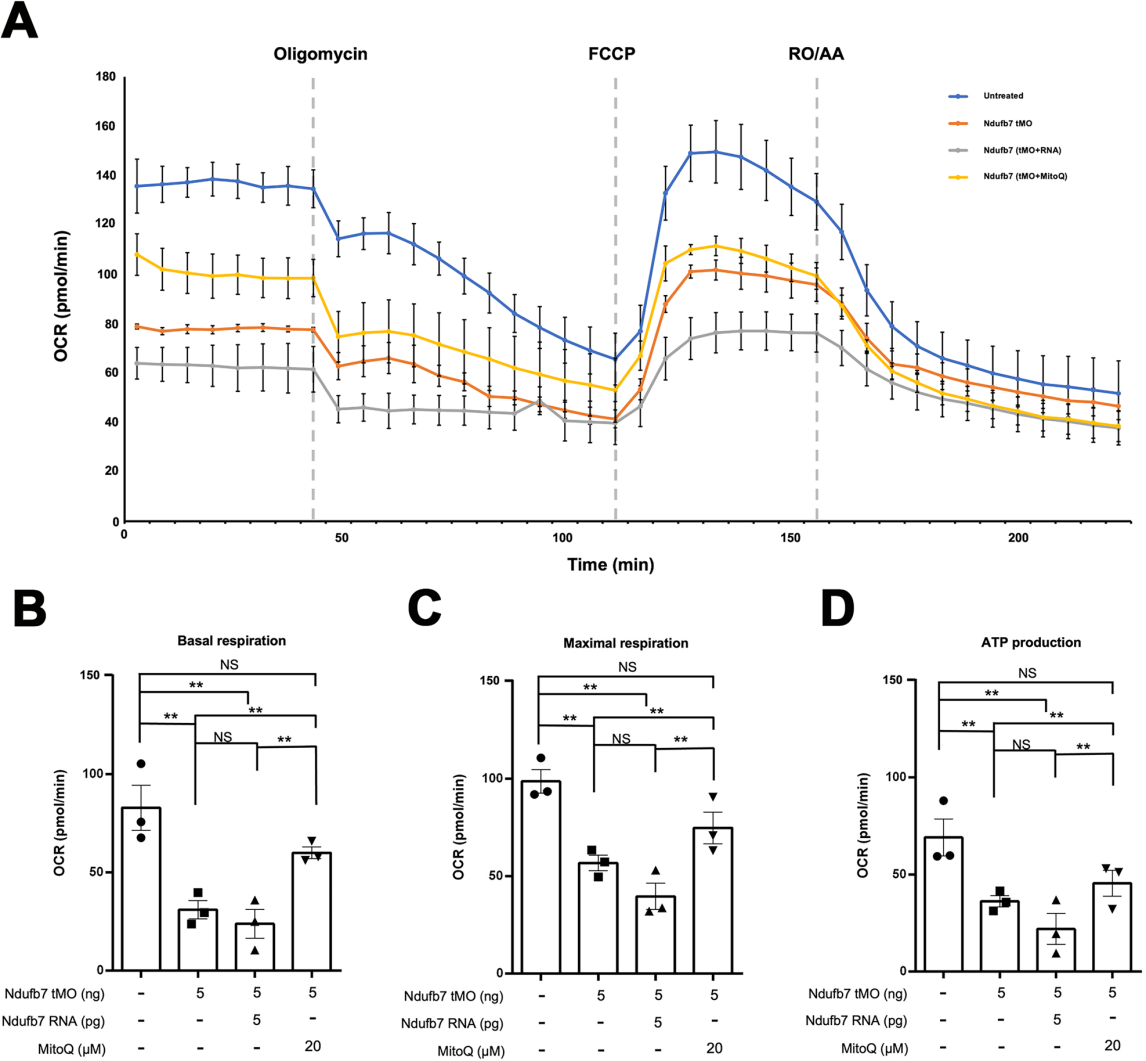
Knockdown of Ndufb7 reduces the oxygen consumption rate. We treated one-cell stage zebrafish embryos without or with indicated Ndufb7 translational-blocking morpholino oligonucleotides (tMO), and Ndufb7 mRNA or Mitoquinone mesylate (MitoQ) as described in Fig. 3, cultured to 24 hours post-fertilization, and subjected them to the measurement of oxygen consumption rate (OCR) using the Seahorse XFe24. **A** Graphical depiction illustrating the changes in mitochondrial respiration upon exposure to oligomycin (18.7 μM), carbonyl cyanide 4-(trifluoromethoxy) phenylhydrazone (FCCP; 8 μM), and rotenone/antimycin A (3.5 μM). In 203 min for each measurement cycle, each inhibitor was injected at a predetermined time point as indicated by a dotted line. The blue, orange, gray, and yellow curves represent the untreated, tMO-injected embryos and tMO with Ndufb7 RNA or MitoQ, respectively. Calculations were made to show **B** Basal respiration, **C** maximal respiration, and **D** adenosine triphosphate (ATP) production. The error bar indicates the standard deviation. Data from three independent experiments are presented, analyzed, and shown as described in Fig. 3. NS not significant; **p < 0.01.

To gain direct insight into the impact of Ndufb7 deficiency in mitochondria respiration, we placed untreated embryos or Ndufb7-morphants at 24 hpf without or with the treatment of Ndufb7 mRNA or MitoQ individually in a 24-well plate to measure the oxygen consumption rate (OCR) under the Agilent Seahorse XFe24 extracellular flux analyzer. We sequentially added various complex inhibitors or decoupling agents of the respiratory chain, specifically oligomycin, carbonyl cyanide-p-trifluoromethoxyphenylhydrazone (FCCP), rotenone, and antimycin A at indicated dosages to examine the ability of mitochondrial energy production by measuring the OCR. As shown in Fig. 7A, the untreated control embryos exhibit higher OCR in each period than other treatments. We calculated and compared the basal respiration rate (Fig. 7B), maximal respiration rate (Fig. 7C), and ATP production (Fig. 7D) between treatments. The Ndufb7-morphants have significantly decreased basal respiration rate, maximal respiration rate, and ATP production, which MitoQ could rescue. Unexpectedly, the Ndufb7 mRNA could not rescue the defect of OCR in Ndufb7-morphants.

## Discussion

There are 31 accessory subunits encoded in the nuclear genome, which are crucial for the stability and functional activity of mitochondrial Complex I. The role of NDUFB7 in mitochondrial stability and activity is not known until recently. Correia et al. identified a homozygous intronic mutation in the NDUFB7 gene (c.113-10C>G) in a patient with severe congenital lactic acidosis and hypertrophic cardiomyopathy [21]. Here, we present a patient with compound heterozygous mutations inherited from healthy parents, exhibiting lactic acidosis, encephalopathy, and abnormal electron transfer chain activity, consistent with mitochondrial disease and resembling the characteristics of a previously reported case. Unlike the previously reported patient who died 55 days after birth from cardiorespiratory failure, our patient, after multiple surgeries and ongoing treatment with CoenzymeQ and vitamin B complex, is now 20 years old and in relatively stable condition. This allows us to experiment, build animal models, and screen for further therapeutic strategies.

We demonstrated that the s patient’s skin fibroblasts are deficient in Complex I protein, which impedes supercomplexes’ formation. In preliminary trials, we observed that the knockdown of NDUFB7 expression by small hairpin RNAs (shRNAs) in human embryonic kidney 293T (HEK293T) cells reduces electron transport chain activities. Furthermore, isolated mitochondria from the patient with the c.113-10C>G mutation showed significantly reduced mitochondrial Complex I enzyme activities [21]. All evidence supports the pathological role of NDUFB7 in mitochondrial diseases.

The accessory proteins provide a scaffold to link the peripheral arm and the distal proton-pumping module of Complex I in the membrane [8]. NDUFB7 is an 18-kDa protein located on a protuberance of a Complex I P module that faces the inter-membrane space (IP1) and is close to ND5 [12, 34**–**36]. At the same time, other reported mutated accessory proteins are all located on the N or Q modules [8, 34]. In a cell line knockout model, decreased Complex I activity has been reported [12]. From the cryogenic electron microscopy map of the I + III_2_ + IV supercomplex (EMD_5319; http://emsearch.rutgers.edu/atlas/5319_summary.html), NDUFB7 in Complex I and cytochrome c in Complex III face the cristae lumen and are close to each other [37, 38]. We speculate that the defect of NDUFB7 causes severe damage to the formation of the membrane arm (P module), and thus, the Q module cannot be assembled with the P module. As a result, the soluble subassembly containing NDUFS3 can be seen. The N module cannot be added in the last stage of the assembly process without the intermediate complex consisting of the P and Q modules. Complex I, III, and IV activities are low in our patient. Mutations of other Complex I accessory protein genes, such as *NDUFS2* and *NDUFS4*, also cause decreased Complex III activity [8].

The current patient also had prenatal-onset growth deficiency, incomplete closure of the abdominal wall (ventral hernia), and poor gastrointestinal tract function (oral dysphagia, gastroesophageal reflux, and delayed gastric emptying). One possible explanation for the occurrence of congenital growth deficiency and structural anomalies (especially tissue deficiencies) is an increase in embryonal cell death because NDUFB7 knockdown was associated with an increase in 293T cell apoptosis in a preliminary trial. However, although growth failure is present in approximately 20% of patients with mitochondrial disease [39], only congenital brain malformations have been observed in mitochondrial disease in the past [40]. Nevertheless, it is biologically plausible that mitochondrial dysfunction is involved in the etiology of VACTERL/VATER association (vertebral defects, anal atresia, cardiac defects, tracheoesophageal fistula, renal anomalies, and limb abnormalities) [41]. Because the number of mitochondrial diseases is still increasing after the application of whole-exome sequencing [42], the spectrum of mitochondrial diseases will certainly expand further.

Zebrafish present a good model to simulate Ndufb7 deficiency. Antisense MO is a convenient and effective tool for knocking down targeted genes in zebrafish [43]. In zebrafish embryos injected with antisense MO against *ndufb7*, the size of the midbrain ventricle was reduced, while the size of the hindbrain ventricle increased. We also observed significantly reduced neuronal volume in both the midbrain and hindbrain. In addition, the Ndufb7-morphants had higher lactic acid levels, a major sign of increased anaerobic respiration and defective mitochondrial respiration, as observed in NDUFB7-deficient patients. Furthermore, these defects could be partially rescued by ectopic expression of *ndufb7*, demonstrating that the defects are due to the specific loss of Ndufb7.

More importantly, the defects could be rescued more effectively using the clinically effective drug MitoQ, which has been proven to relieve the patient’s symptoms. Previous research indicates that MitoQ shields the liver from injury by clearing intracellular and mitochondrial reactive oxygen species, maintaining the integrity and functionality of mitochondria, preventing apoptosis, and blocking the release of mitochondrial DNA from Kupffer cells [44]. Animals with heart failure receiving MitoQ showed decreased right ventricular hypertrophy and alleviated lung congestion [45]. Both Ndufb7 RNA and MitoQ can partially rescue brain defects and completely rescue the lactic acid levels in zebrafish embryos. However, only MitoQ, not Ndufb7 RNA, could rescue the defective mitochondrial respiration. The failure of Ndufb7 RNA to rescue might be due to the low translocation efficiency from the nucleus to the mitochondria and the failure in the assembly of Ndufb7 into Complex I.

The prevention of lactic acid accumulation by ectopic expression of *ndufb7* suggests that it may either maintain near-basal mitochondrial activity, thereby blocking unnecessary anaerobic respiration, or play a role in modulating anaerobic respiration in the cytosol.

To our knowledge, only two pathogenic NDUFB7 variants have been reported (this study; Correia et al. [21]), with four additional uncertain significance variants submitted on the ClinVar Miner. The rarity of available cases makes mechanistic analyses and the development of therapeutic strategies extremely challenging. The established Ndufb7-deficient zebrafish model can thus address this unmet need for future studies and potential drug screening.

## Materials and methods

### Brain magnetic resonance imaging

A brain magnetic resonance imaging (MRI) scan was performed using a standard birdcage head coil on a 1.5-T Sonata MRI system (Siemens Healthineer, Forchheim, Germany) [46**–**48]. During the examination, the patient was sedated. Standard methods were employed to acquire T2-weighted, fluid-attenuated inversion recovery (FLAIR) images with a slice thickness of approximately 4–5 mm.

### Electron transfer chain activity assay

Skin fibroblast electron transfer chain activities were measured according to previous reports [49**–**51]. In brief, the enzyme activities of NADH: Ferricyanide dehydrogenase (Complex I), NADH: Cytochrome c reductase (Complex I + III) (total and rotenone-sensitive), succinate dehydrogenase (Complex II), cytochrome c oxidase (Complex IV), and citrate synthase were determined by measuring changes in optical density using a spectrophotometer (TECAN) after the addition of various substrates. The experiment was performed in triplicate.

### Whole-exome sequencing

The whole-exome sequencing was done at Radboudumc University Medical Center, Nijmegen, the Netherlands. Coding DNA sequencing was enriched with an Agilent SureSelectXT Human All Exon 50 Mb kit (version V5+UTRs; Agilent Technologies, CA, USA) and was performed in an HiSeq 2000^TM^ sequencer (Illumina, San Diego, CA, USA), aligned by Burrows-Wheeler Aligner (BWA; GRCh37) [52] and subjected to variant calling by GATK (Broad Institute, Cambridge, MA, USA) in BGI-Europe (Copenhagen, Denmark).

### Biochemical analyses of mitochondrial assembly and supercomplex

Fibroblasts were cultured from the patient skin biopsy. One-dimensional blue native polyacrylamide gel electrophoresis (BN-PAGE) was performed with anti-NDUFA9 for Complex I, anti-70 kDa Fp subunit for Complex II, anti-subunit core 1 for Complex III, and anti-subunit I for Complex IV [53]. Triton X-100 was used as a detergent to separate the individual complexes, and digitonin was used as a gentle detergent to observe the formation of supercomplexes. A second dimension of denaturing discontinuous gel electrophoresis was added in two-dimensional BN-PAGE. Antibodies were used to identify specific proteins, including NDUFV1 in the N module, NDUFS3 in the Q module, and NDUFB10 in the membranous P module. Skin fibroblast electron transfer chain activities were measured according to previous reports [49**–**51]. Antibodies used as follows: NDUFA9 (Invitrogen, Catalog # 459100); 70 kDa Fp subunit (Invitrogen, Catalog # 459200); Subunit Core 1 (Invitrogen, Catalog # 459140); Subunit 1 (Invitrogen, Catalog # 459600); NDUFS3 (Abnova, Catalog # H00004722-A01); NDUFB10 (Abnova; Catalog # H00004716-A01); NDUFV1 (Proteintech, Catalog # 11238-1-AP).

### Zebrafish

Wild-type AB zebrafish (*Danio rerio*) sourced from the Taiwan Zebrafish Core Facility at Academia Sinica, Taiwan, were housed at a temperature of 28.5 °C under a light cycle of 14 h light and 10 h dark. Embryos obtained from natural mating of fish aged 4-12 months were cultured in Danieau’s buffer (17.4 mM NaCl, 0.21 mM KCl,0.12 mM MgSO_4_, 0.18 mM Ca(NO3)_2_, 1.5 mM HEPES, pH 7.3) supplemented with 1.54 μΜ methylene blue (Hsin Kung Pharmaceutical MFG, Tainan, Taiwan).

### Microinjection

Antisense morpholinos designed against *ndufb7* (tMO: CAAGATGAGCGCCCATCCCGATC) were synthesized by Gene Tools (Philomath OR, USA). *Ndufb7* RNA for injection was prepared using the HiYield^TM^ plasmid Kit Mini (Arrowtech, catalog number (Cat#) AHP-300, Taipei, Taiwan) and mMESSAGE mMACHINE^TM^ SP6 (Invitrogen, Cat# AM1340, Waltham MA, USA). We injected an embryo with 2.3 nL solution with 50% Phenol red solution (Sigma Aldrich, Cat # P0290). The microinjection solution contains 5 ng of Ndufb7 tMO without or with 5 pg Ndufb7 RNA or 20 µM Mitoquinone mesylate (MitoQ, MedChemExpress, Cat# HY-100116A, Monmouth Junction, NJ, USA) to rescue the Ndufb7 tMO-induced defects. All reagents were injected at the 1-cell stage, and embryos were raised to 24 or 48 hpf at 28.5 °C.

### Measurement of brain ventricle area

Embryos under different treatments were treated with 0.003% 1-phenyl 2-thiourea (*PTU*) (Sigma Aldrich, Cat# P7629, St. Louis, USA) at 24 hpf, preventing the pigment from interfering with the observation of the brain ventricle, raised to 48 hpf, and photographed under brightfield of a stereo-fluorescent microscope (SZX7, Olympus, Tokyo, Japan). The area of brain ventricles was measured using the Image J software to count the pixels in both the midbrain and hindbrain ventricles. Alternatively, we injected dextran, rhodamine B (Invitrogen, Cat# D1824) into brain ventricles of 48-hpf larvae with different treatments, photographed under darkfield, and the areas of dextran rhodamine-filled ventricles were measured and analyzed as described above.

### Measurement of neuronal volume in the midbrain and hindbrain

Differently treated Tg(*Huc:kaede*) zebrafish embryos were depigmented with PTU as described, raised to 48 hpf, observed, and imaged using an LSM 780 confocal laser-scanning microscope (Carl Zeiss, Oberkochen, Germany). We scanned the midbrain in the lateral view with stacks composed of 25 10 μm-thick layers. The volumes of brain neurons were analyzed by the MetaMorph software 4D viewer (Molecular Devices, San Jose, CA, USA).

### Measurement of lactic acid

We collected 80–100 embryos in a 1.5-mL Eppendorf tube with 300 µL 0.1%NaCl. We homogenized the samples with a BRANSON digital signifier (Branson, Brookfield, CT, USA) for 5 min at 20% amplitude, for 2 seconds at a time with 3 seconds apart. Then, we centrifuged the homogenates at 10,000 × *g* for 10 minutes to collect the supernatant. Protein concentration was measured by the Pierce™ BCA Protein Assay Kits (Thermo Scientific, Cat# 23225, *Waltham MA*, USA), and lactic acid content was measured by the L-Lactic Acid (LA) Colorimetric Assay Kit (Elabscience, Cat# E-BC-K044-S, Houston TX, USA)

### Seahorse XFe24 cell Mito stress assay

The oxygen consumption rate (OCR) of 24- hpf zebrafish embryos was measured using a Seahorse XFe24 extracellular flux analyzer (Agilent, *Santa Clara, CA*, USA). The temperature was maintained at 28.5 °C. Embryos were placed individually in wells of a Seahorse XFe24 Islet Capture FluxPak (Agilent, Cat#103518-100 *Santa Clara, CA*, USA) containing 525 μL of Danieau’s buffer, and processed as reported [54]. A grid was used to keep the larvae at the bottom of the wells throughout experiments. Four wells per experiment were kept empty to serve as the “blank” condition. At the 1-cell stage, an embryo was injected with 5 ng tMO without or with 5 pg Ndufb7 RNA or 20 µM MitoQ, and raised to 24 hpf. The OCR measurements were conducted following the manufacturer’s instructions. Eight basal analysis cycles were recorded, followed by 12 cycles after the administration of 18.7 μM oligomycin (MedChemExpress, Cat# HY-N26782), 8 cycles after the administration of 8 μM carbonyl cyanide-p-trifluoromethoxyphenylhydrazone (FCCP, MedChemExpress, Cat# HY-100410), and 12 cycles after administration of 3.5 μM rotenone (Sigma Aldrich, Cat# R8875) and 3.5 μM antimycin A (Sigma Cat# A8674). OCR was calculated for basal, maximal, and ATP production phases.

### Statistical analyses

For all quantitative data, each dot represents one embryo. All experimental values are presented as mean ± standard error of the mean (SEM) and were analyzed by the Whitney U test. The number at the bottom or above the bar indicates the total sample number of the experiment. Data from at least three independent experiments are presented and analyzed. NS: not significant; *p < 0.05; **p < 0.01; ***p < 0.001; ****p < 0.0001.

### Web resources

AlphaFold: https://alphafoldserver.com/. ClinVar Miner: https://clinvarminer.genetics.utah.edu/. dbSNP: http://www.ncbi.nlm.nih.gov/projects/SNP/. Exome Aggregation Consortium (ExAC) Browser: http://exac.broadinstitute.org/. NHLBI Exome Sequencing Project (ESP) Exome Variant Server: http://evs.gs.washington.edu/EVS/. Polyphen-2: http://genetics.bwh.harvard.edu/pph2/. PROVEAN: http://provean.jcvi.org/. RefSeq: http://www.ncbi.nlm.nih.gov/RefSeq. RCSB protein data bank: http://www.rcsb.org/. SIFT: http://sift.jcvi.org/.

## Supplementary information

Wetsern blots

Supplementary Figure 1. Structural prediction and alignment of NDUFB7 and its variants

Supplementary video 1

Supplementary Video 2

## Data Availability

The dataset generated and/or analyzed during the current study is available from the corresponding author on request.
