# Supplementary material for: NDUFB7 mutations cause brain neuronal defects, lactic acidosis, and mitochondrial dysfunction in humans and zebrafish
Source: Cell Death Discov. 2025 Mar 1;11:82. doi: 10.1038/s41420-025-02369-0 (PMC11873233; doi:10.1038/s41420-025-02369-0)

Western blot of Figure 2A
Complex I (NDUFA9)

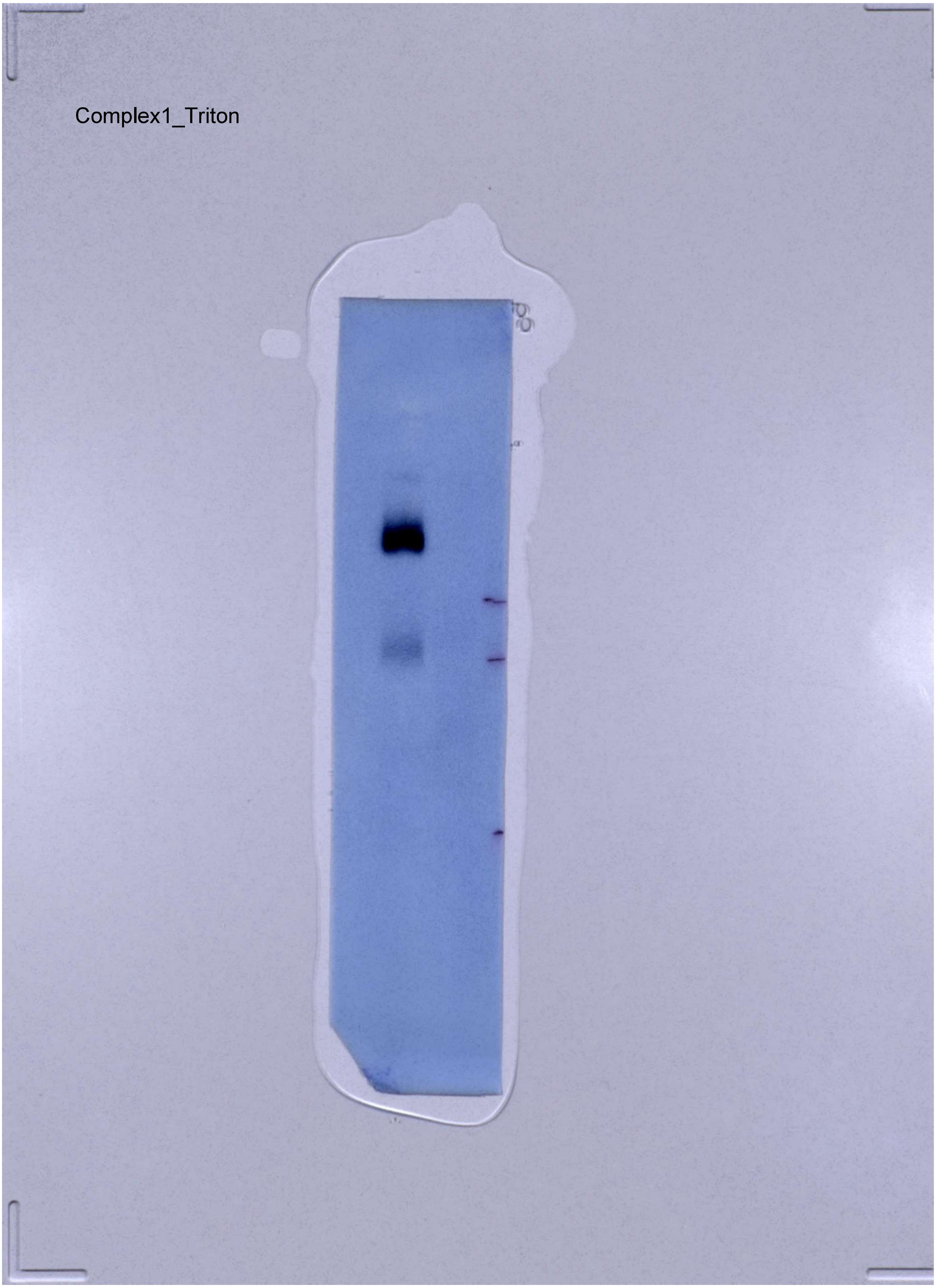

Western blot of Figure 2A
Complex II for complex I lanes

Complex2_Triton_Complex1membrane

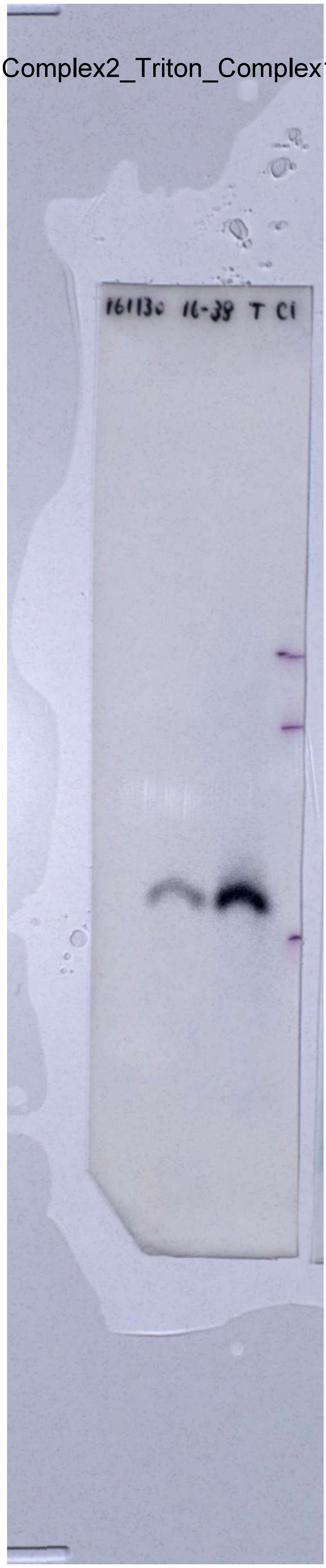

Western blot of Figure 2A
Complex II (70kDa Fp Subunit)

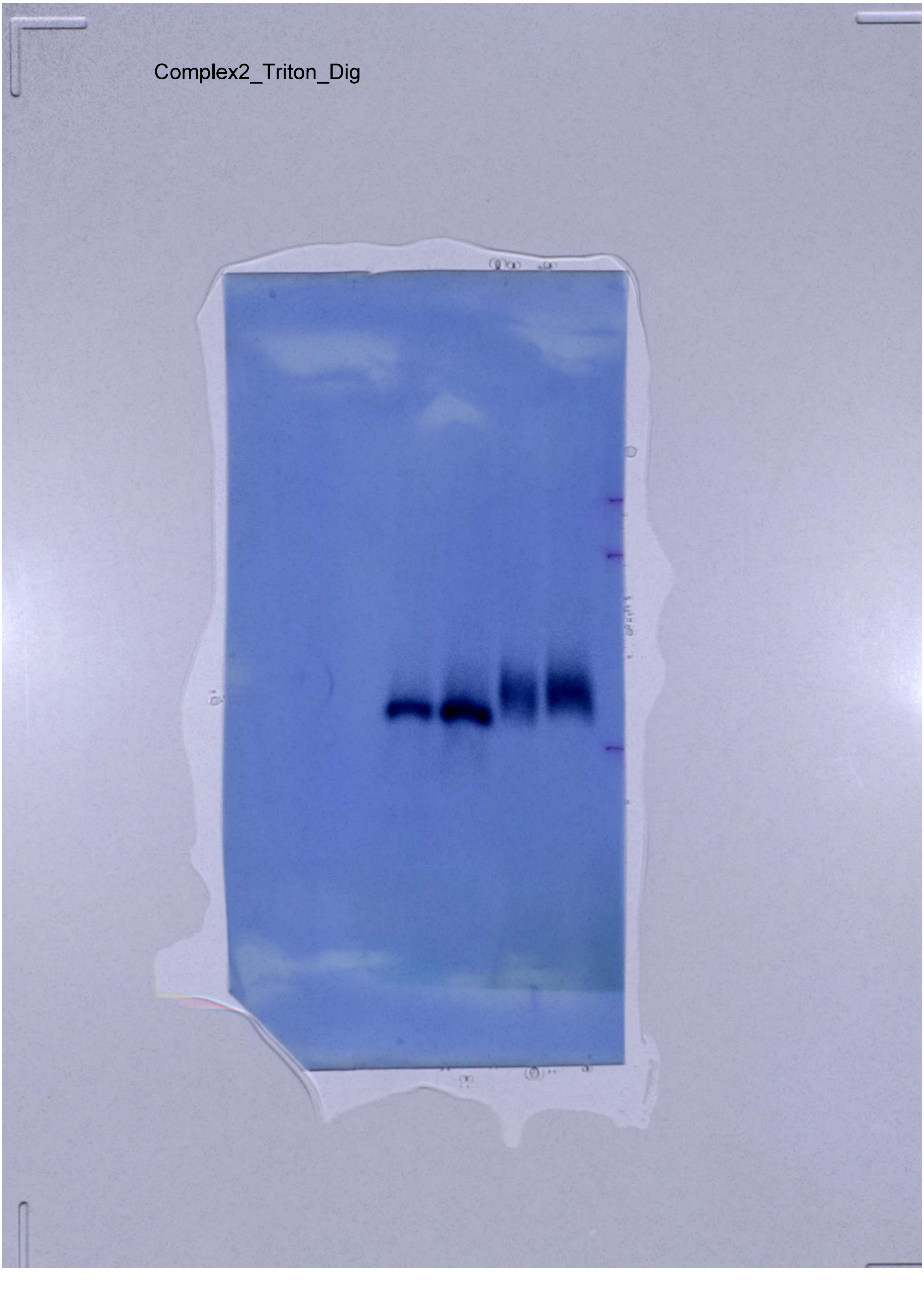

Western blot of Figure 2A
Complex III (Subunit core 1)

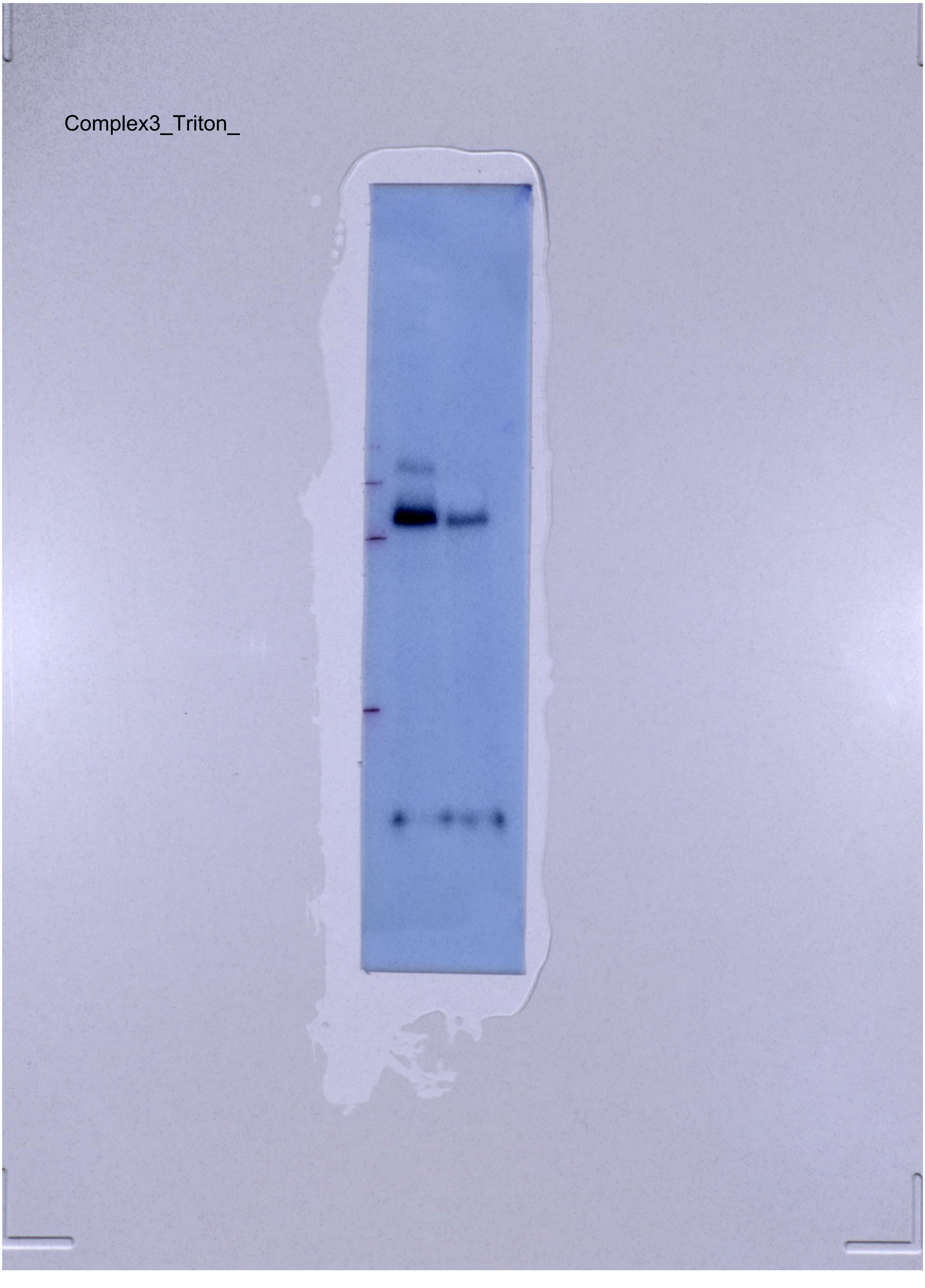

Western blot of Figure 2A
Complex II for Complex III lanes

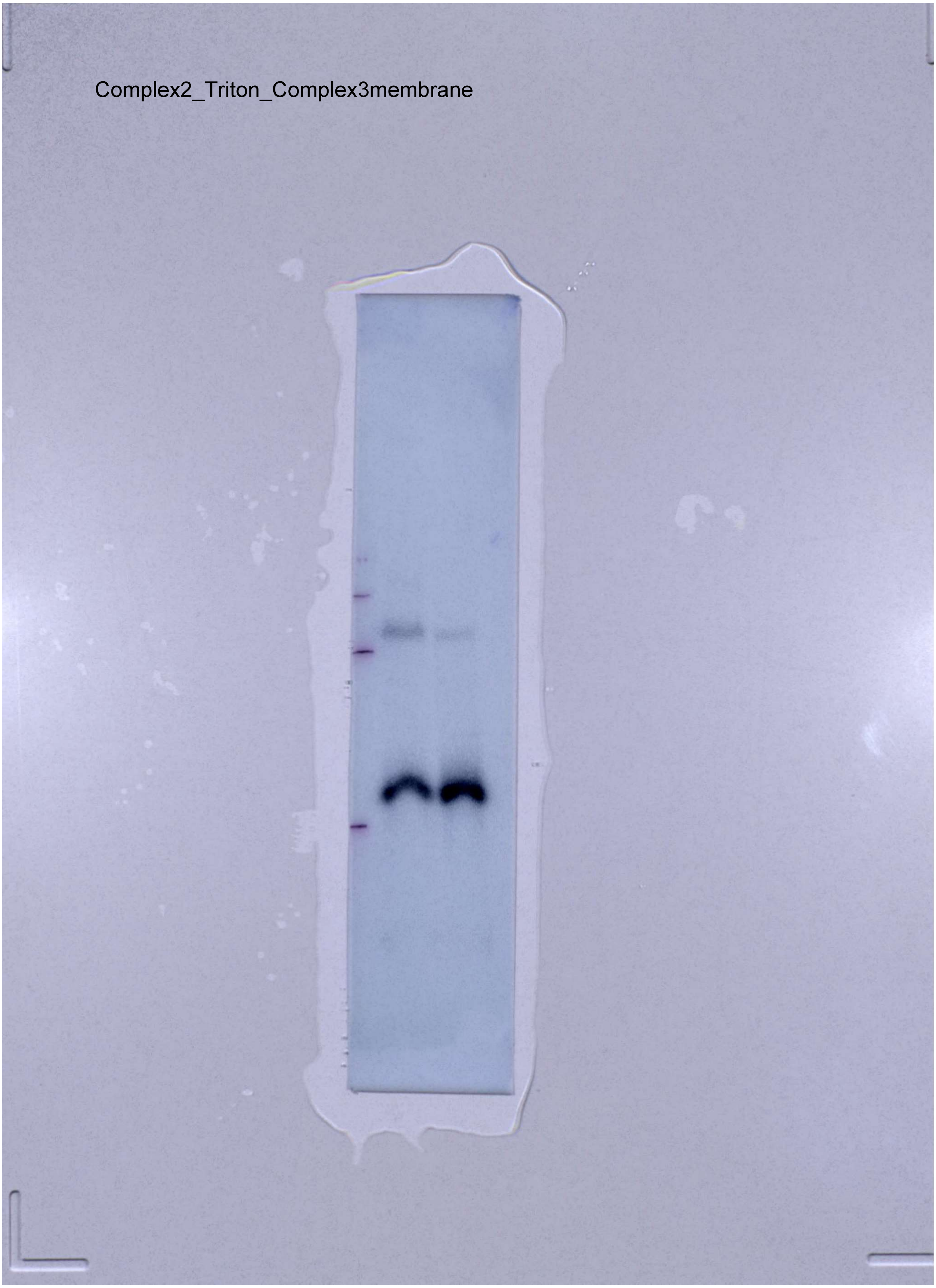

Western blot of Figure 2A
Complex IV (subunit I)

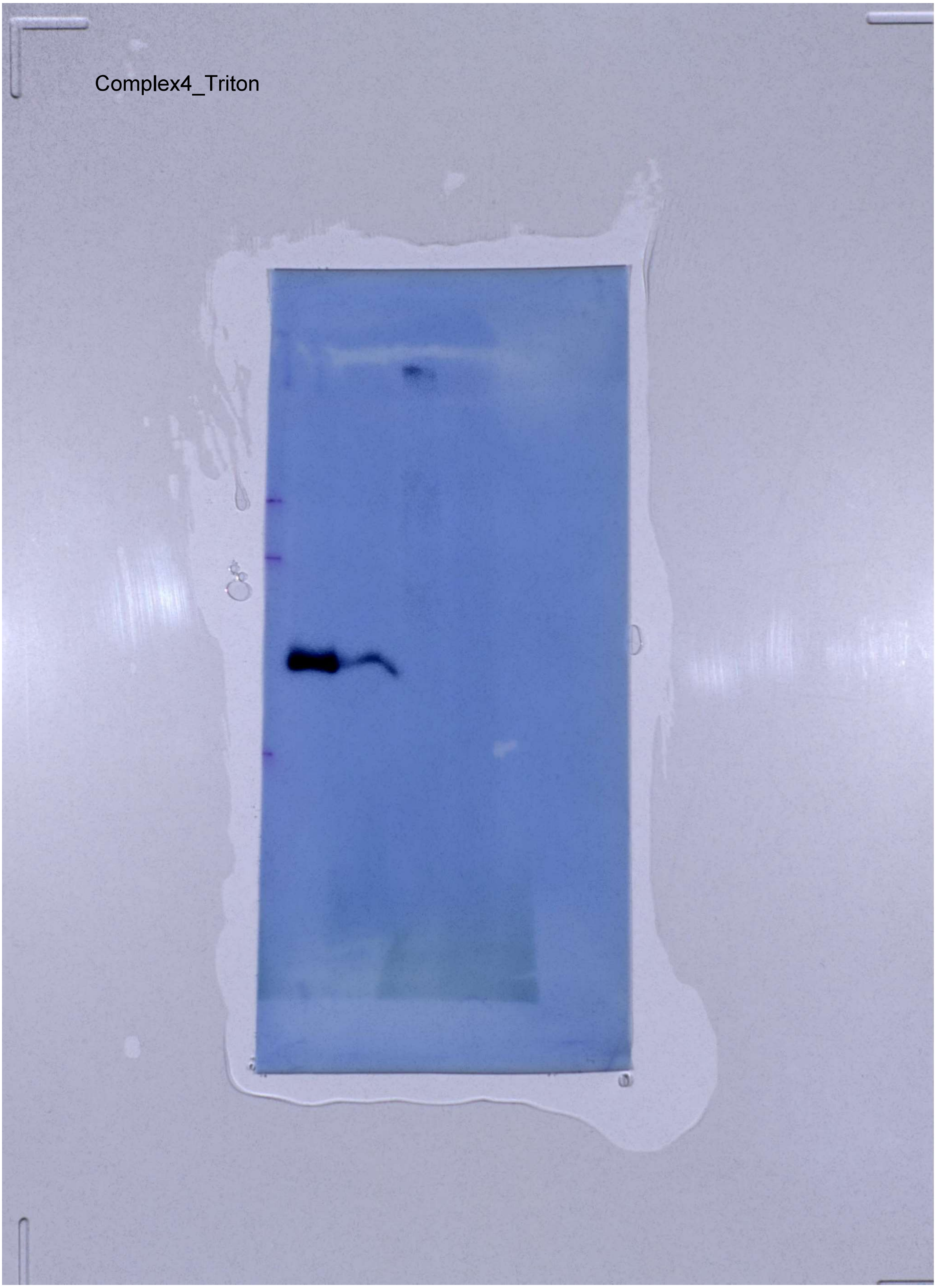

Western blot of Figure 2A
Complex II for Complex IV lanes

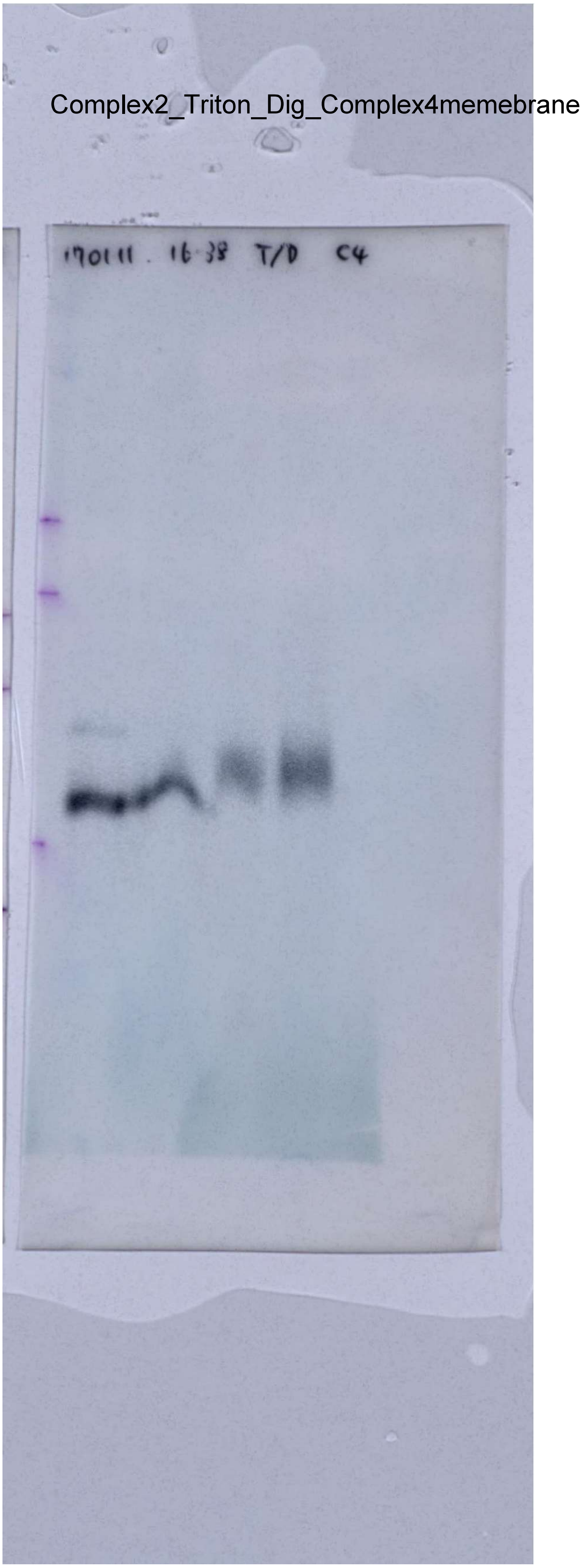

Western blot of Figure 2B
Complex I (NDUFA9)

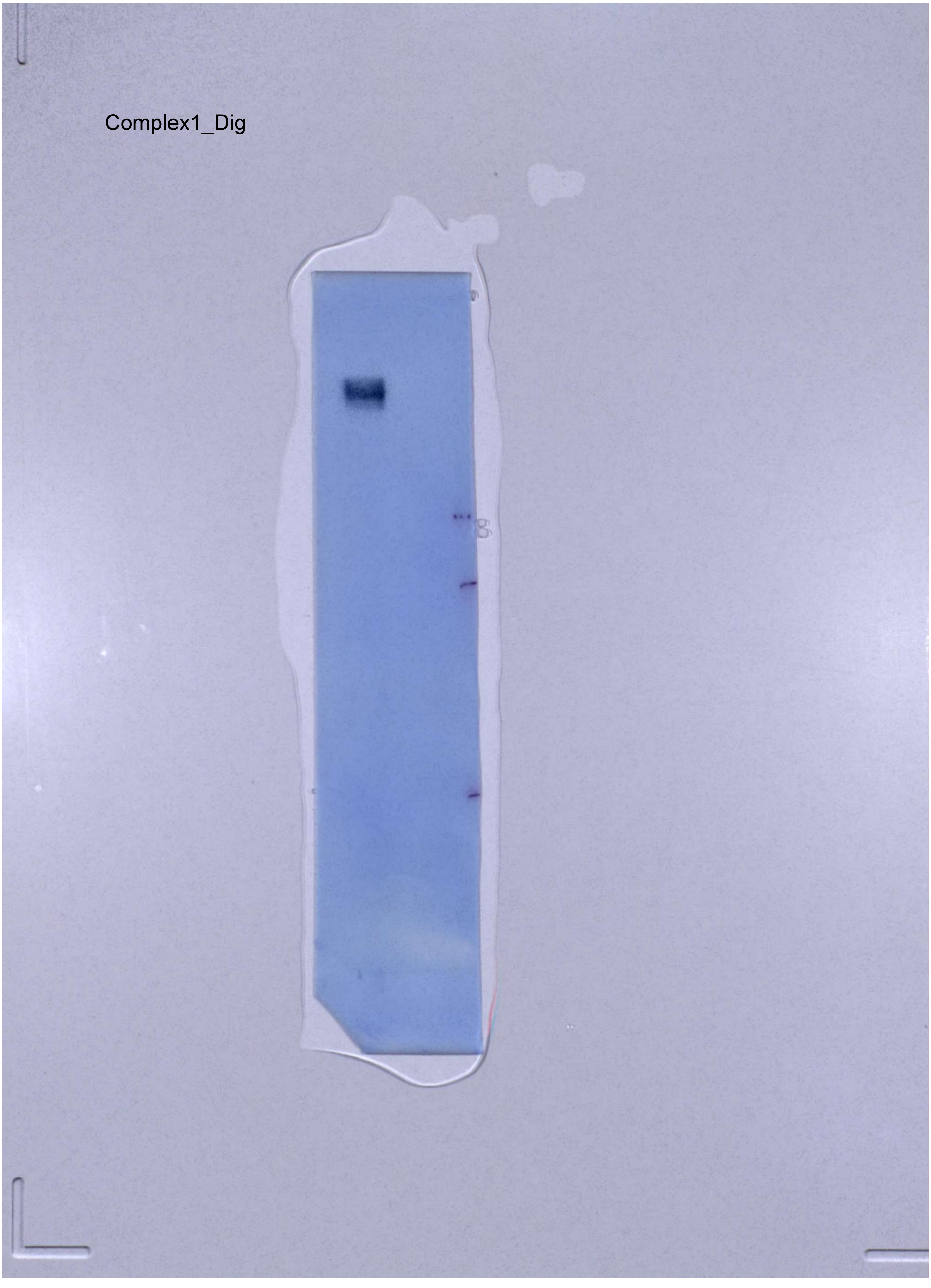

Western blot of Figure 2B
Complex II for Complex I lanes

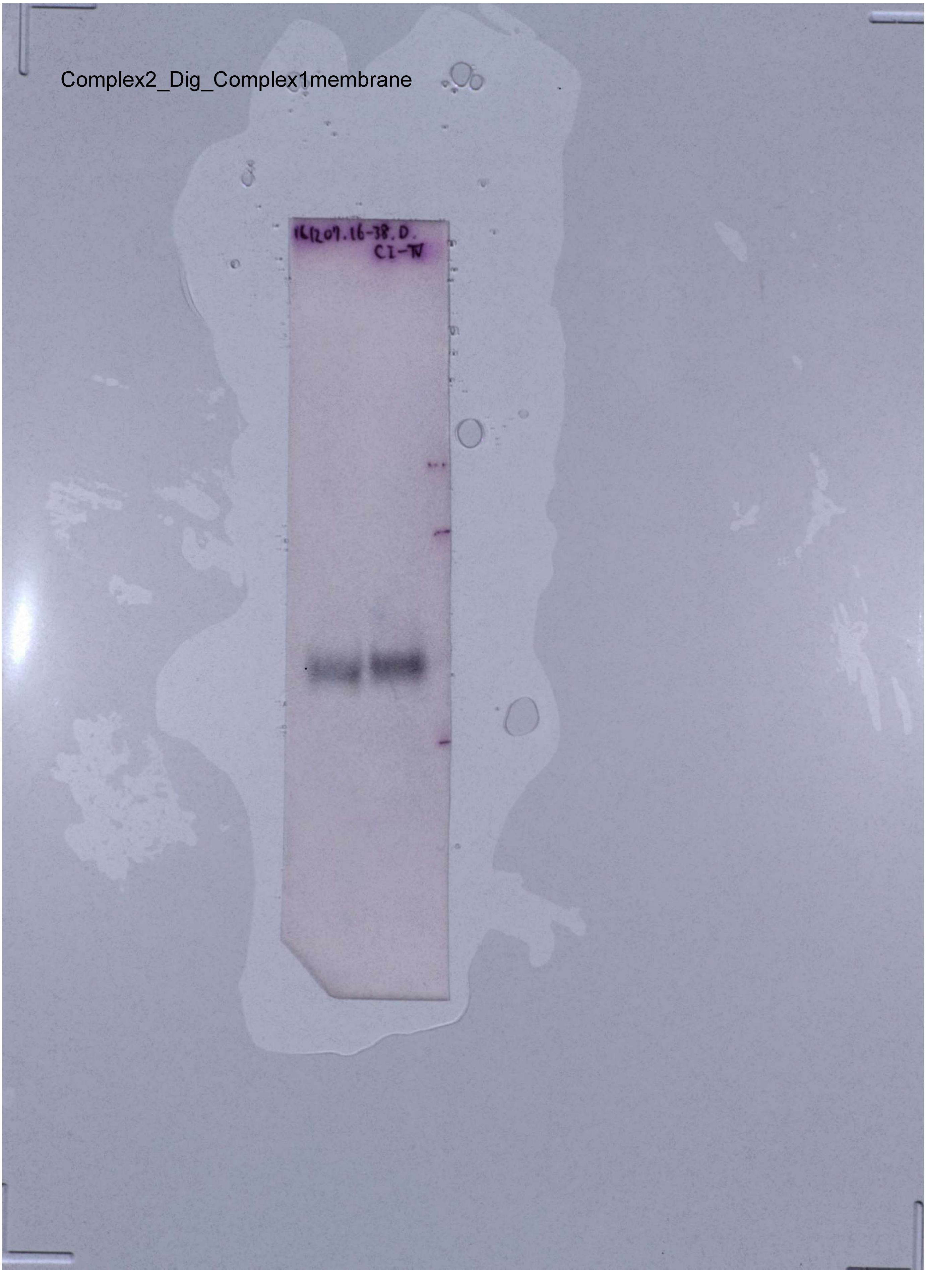

Western blot of Figure 2B
Complex II (70kDa Fp Subunit)

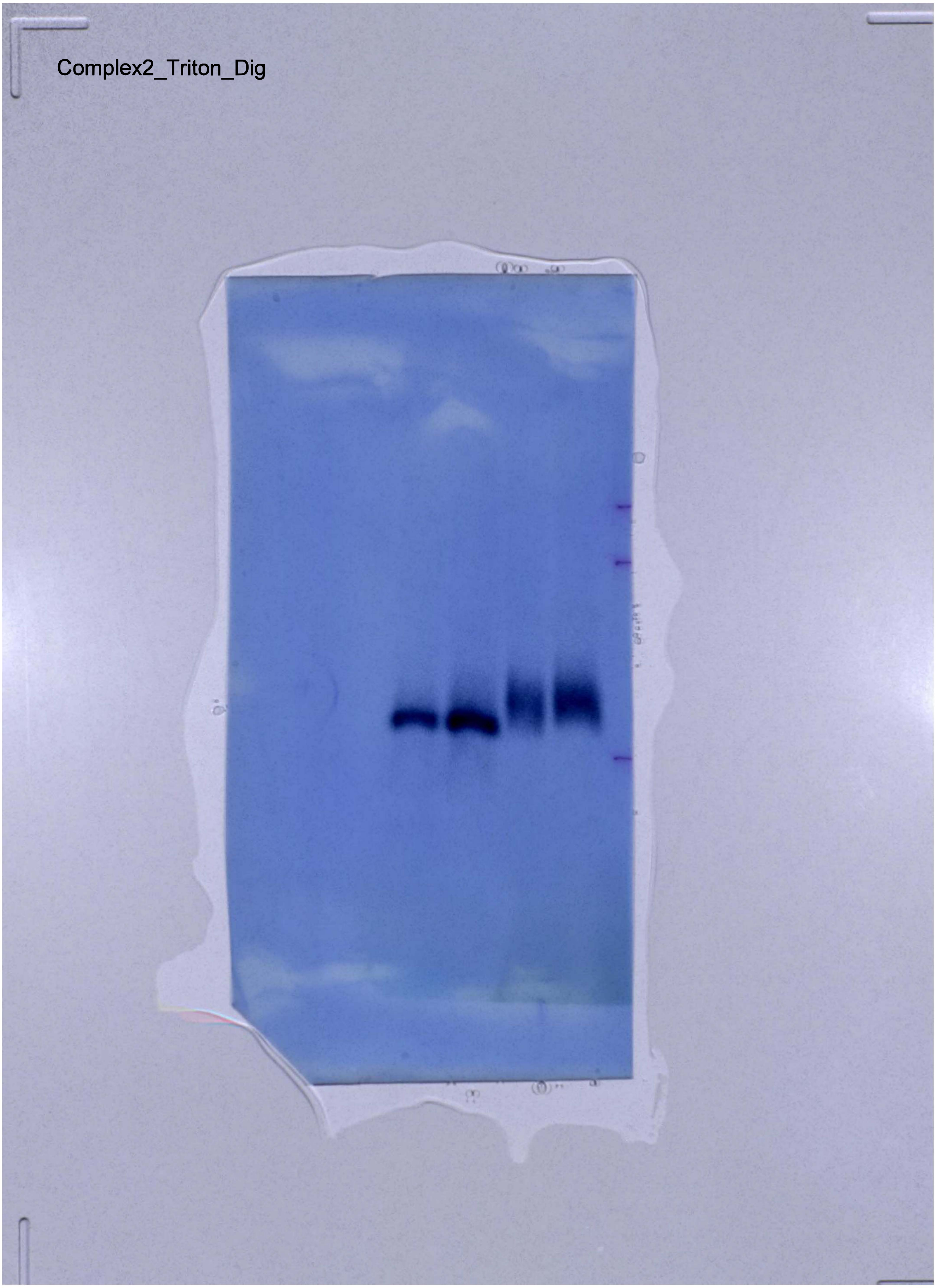

Western blot of Figure 2B
Complex III (subunit core 1)

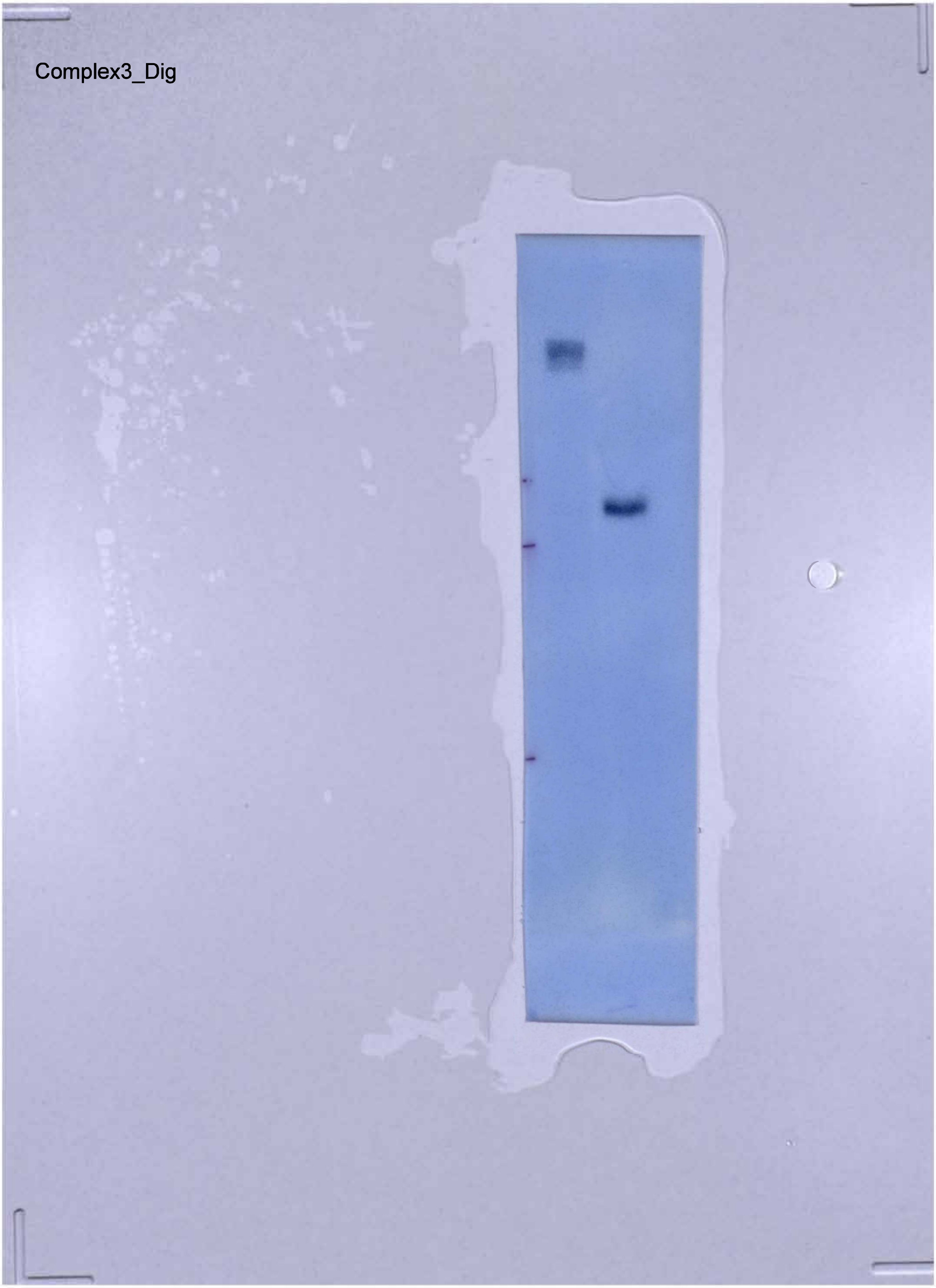

Western blot of Figure 2B
Complex II for complex III lanes

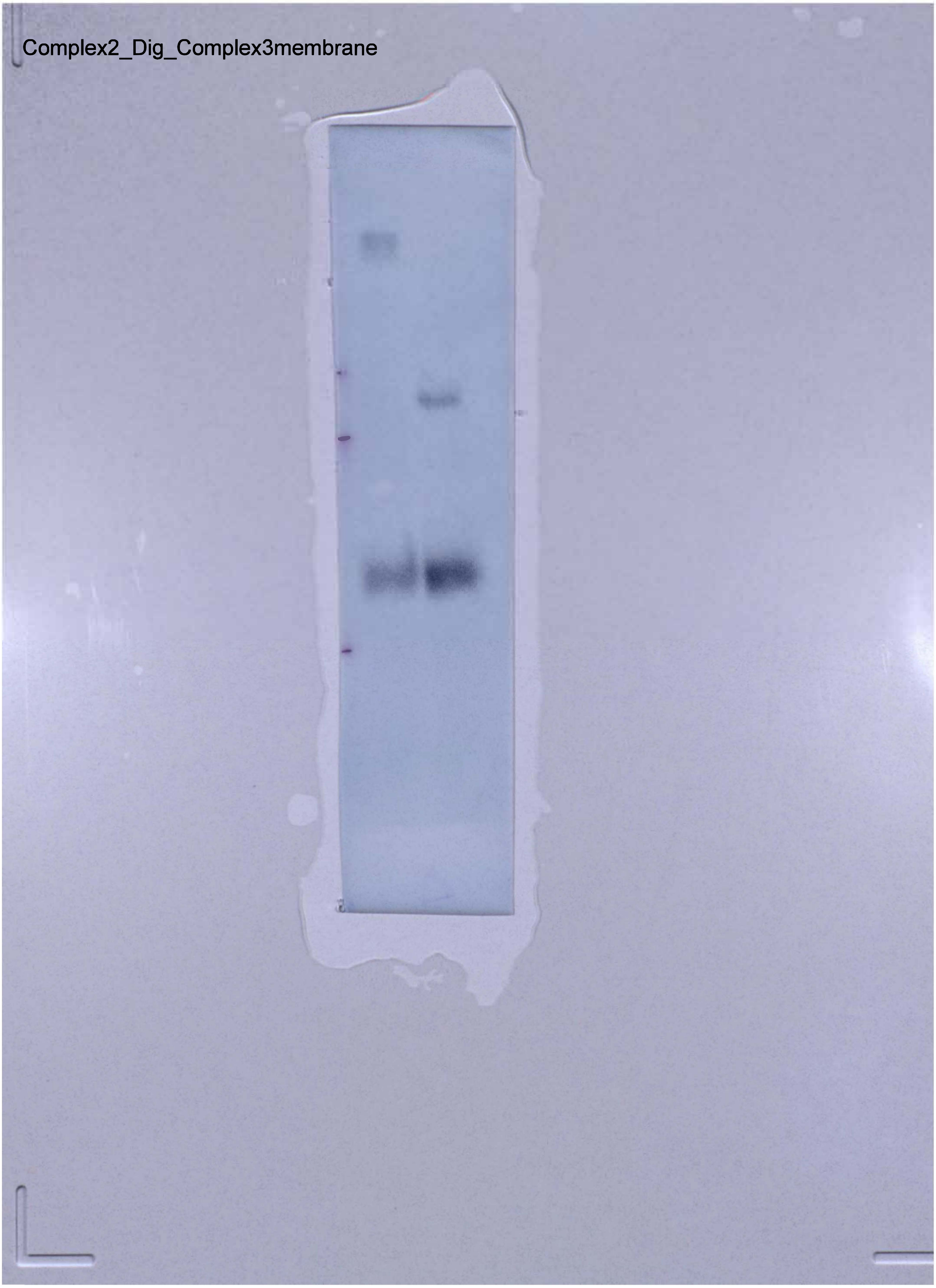

Western blot of Figure 2B
Complex IV (subunit I)

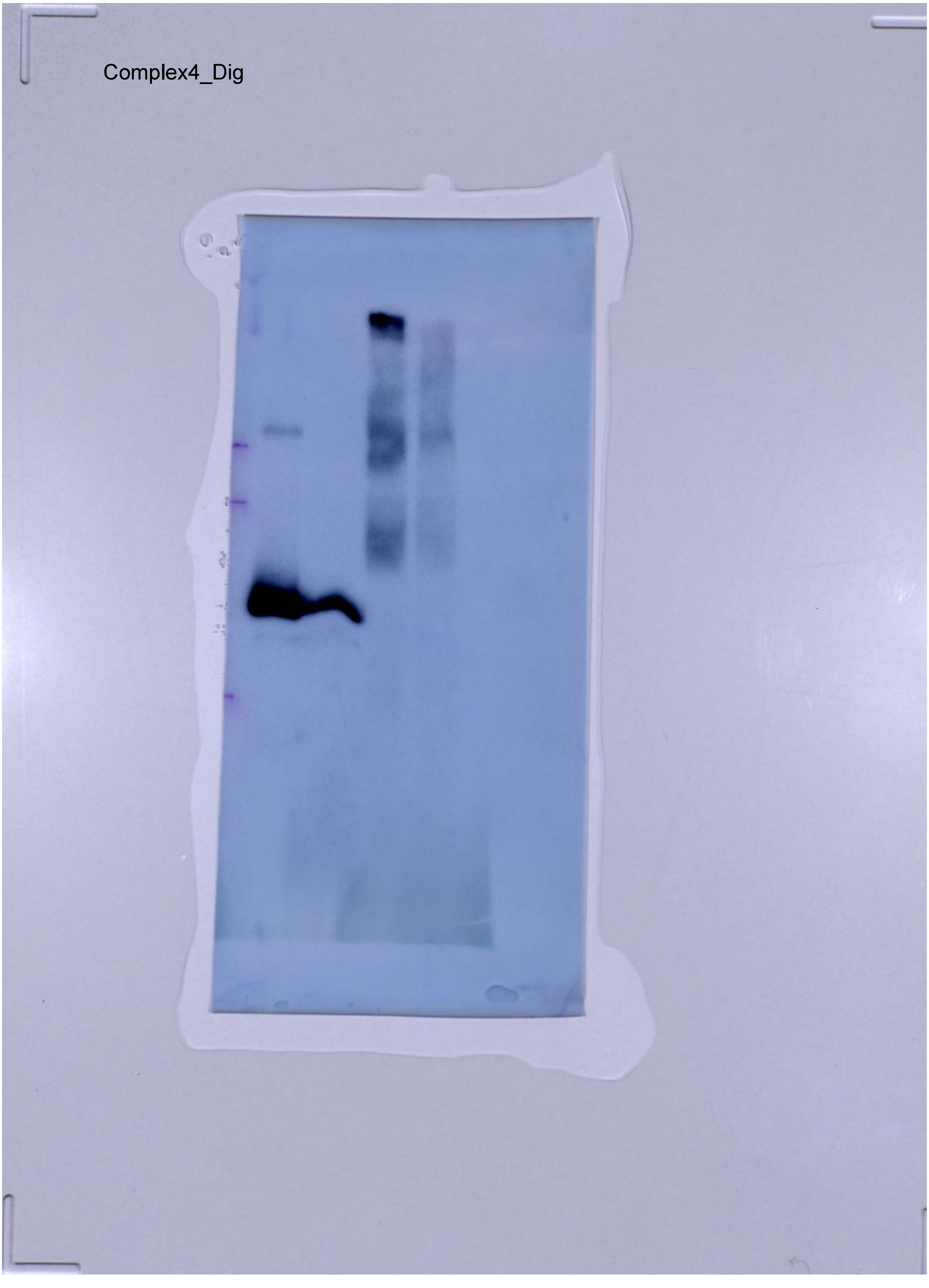

Western blot of Figure 2B
Complex II for complex IV lanes

Complex2_Triton_Dig_Complex4memebrane

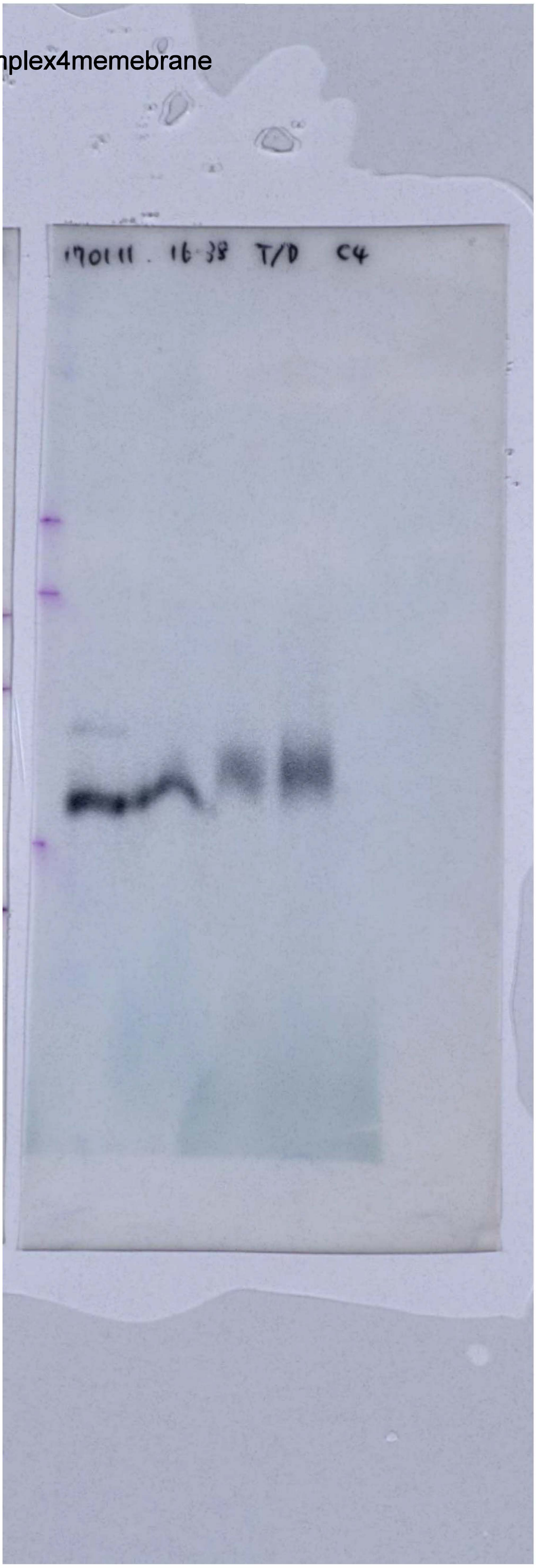

Western blot of Figure 2C
2D:SDS-PAGE of Control_NDUFA9

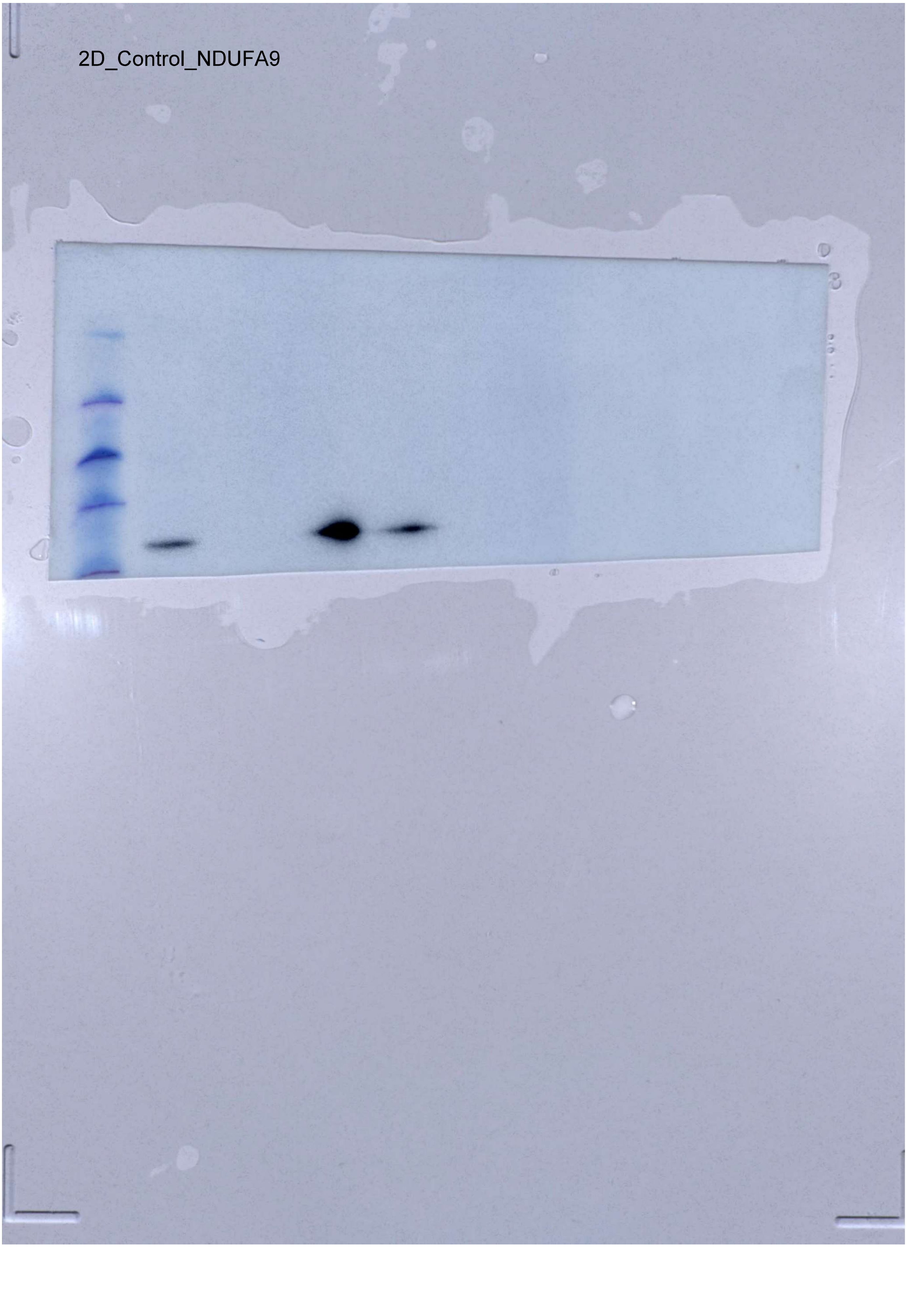

Western blot of Figure 2C
2D:SDS-PAGE of Patient_NDUFA9+NDUFB10

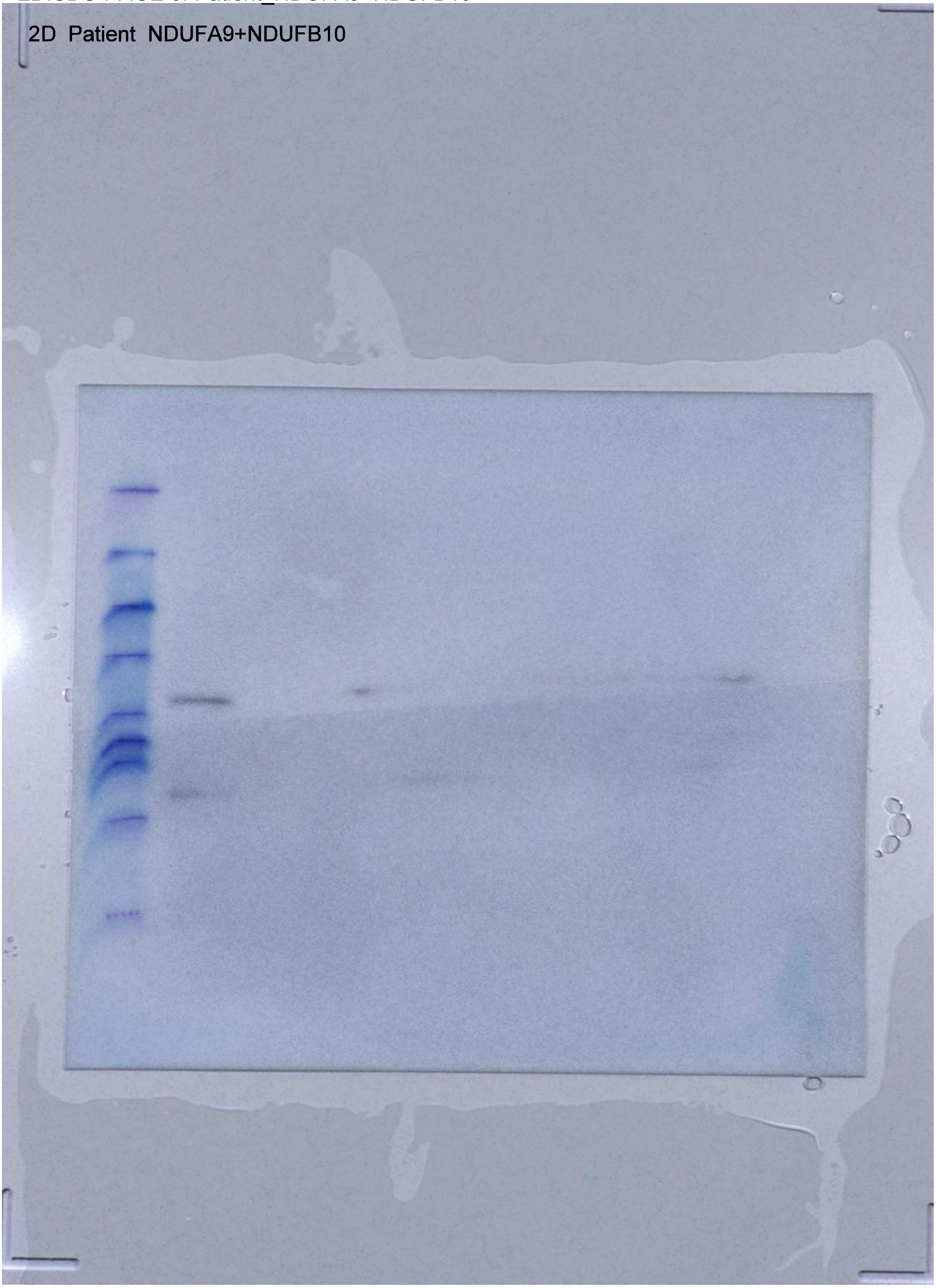

Western blot of Figure 2C
2D:SDS-PAGE of Patient_NDUFS2+NDUFS3

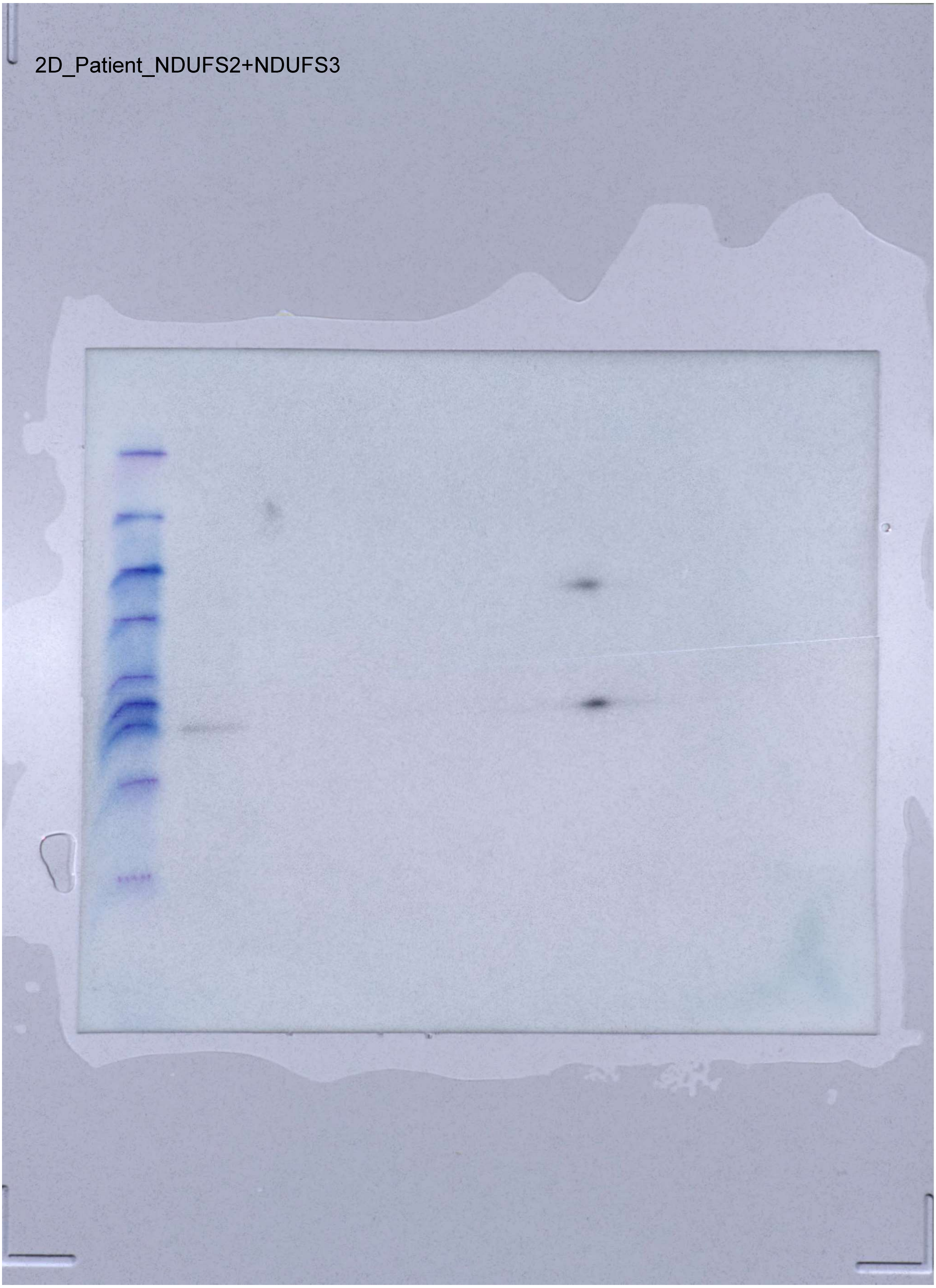

Western blot of Figure 2C
2D:SDS-PAGE of Control_NDUFS3

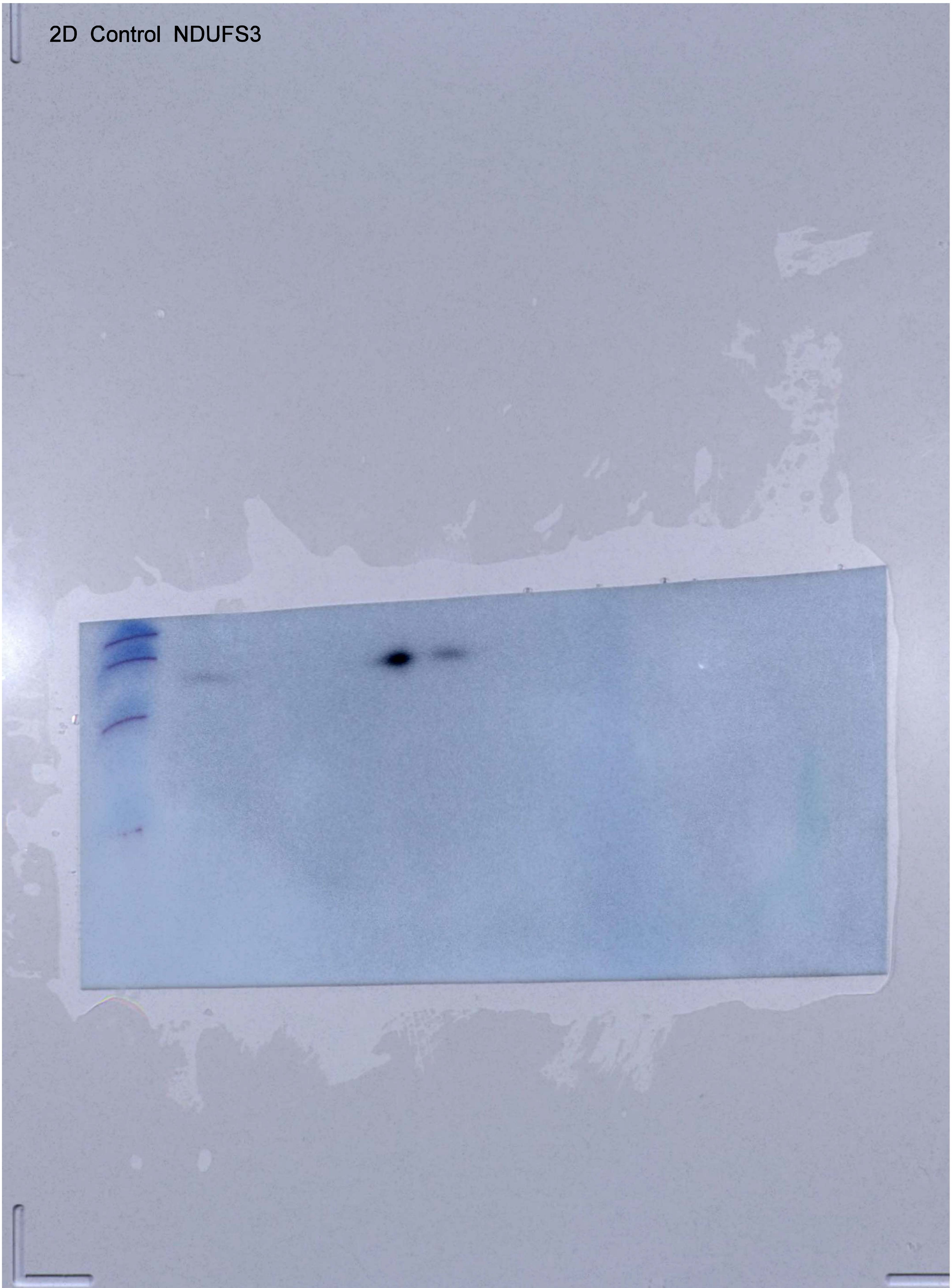

Western blot of Figure 2C
2D:SDS-PAGE of Control_NDUFB10

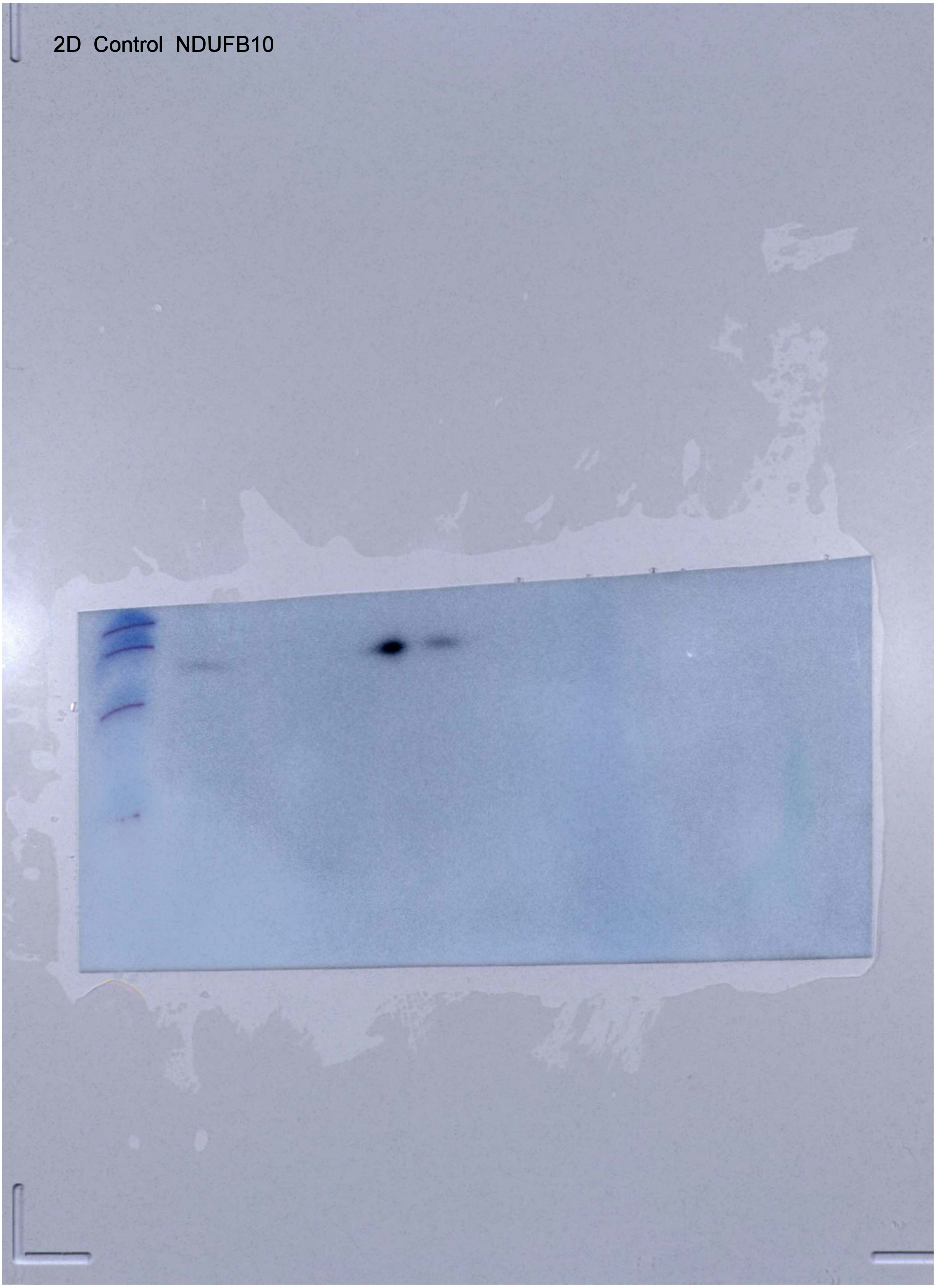

Western blot of Figure 2C
2D:SDS-PAGE of Control and Patient of NDUFV1

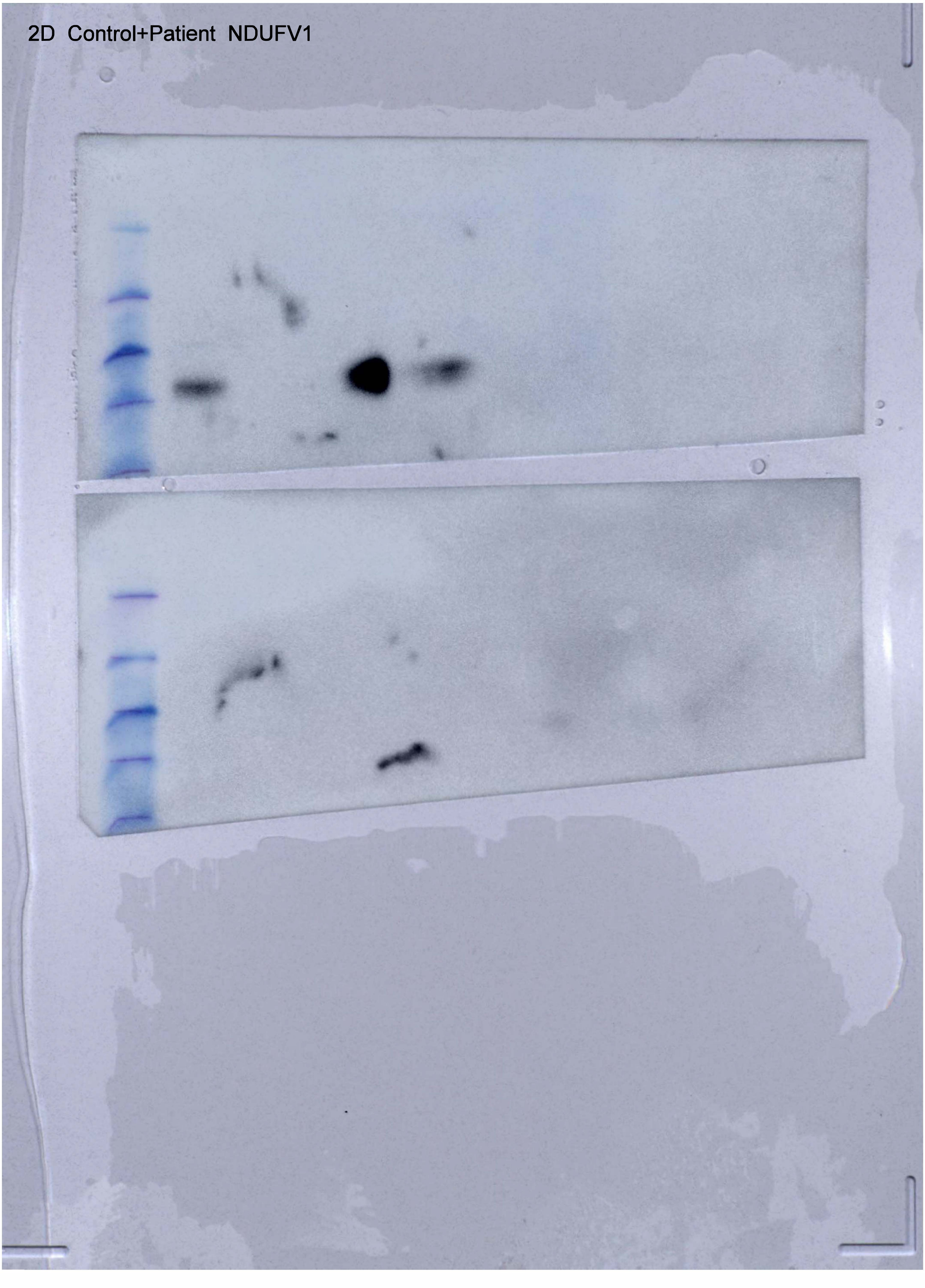

Western blot of Figure 2C
2D:SDS-PAGE of Control and Patient of CII 70kDa

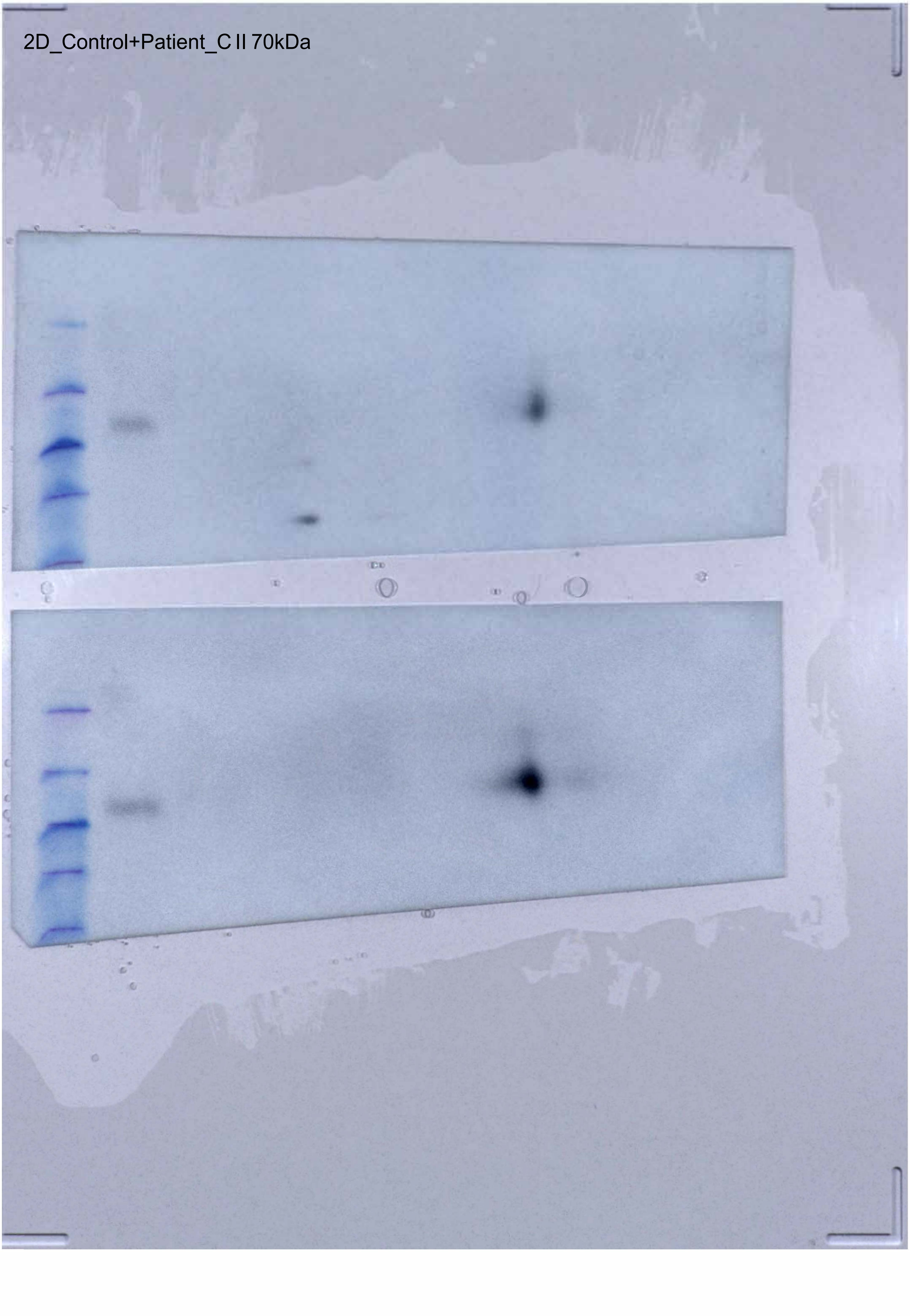

Supplement: Supplementary file 1 — Wetsern blots [file 41420_2025_2369_MOESM1_ESM.pdf]
