# Supplementary material for: NDUFB7 mutations cause brain neuronal defects, lactic acidosis, and mitochondrial dysfunction in humans and zebrafish
Source: Cell Death Discov. 2025 Mar 1;11:82. doi: 10.1038/s41420-025-02369-0 (PMC11873233; doi:10.1038/s41420-025-02369-0)
Supplement: Supplementary file 2 — Supplementary Figure 1. Structural prediction and alignment of NDUFB7 and its variants [file 41420_2025_2369_MOESM2_ESM.docx]

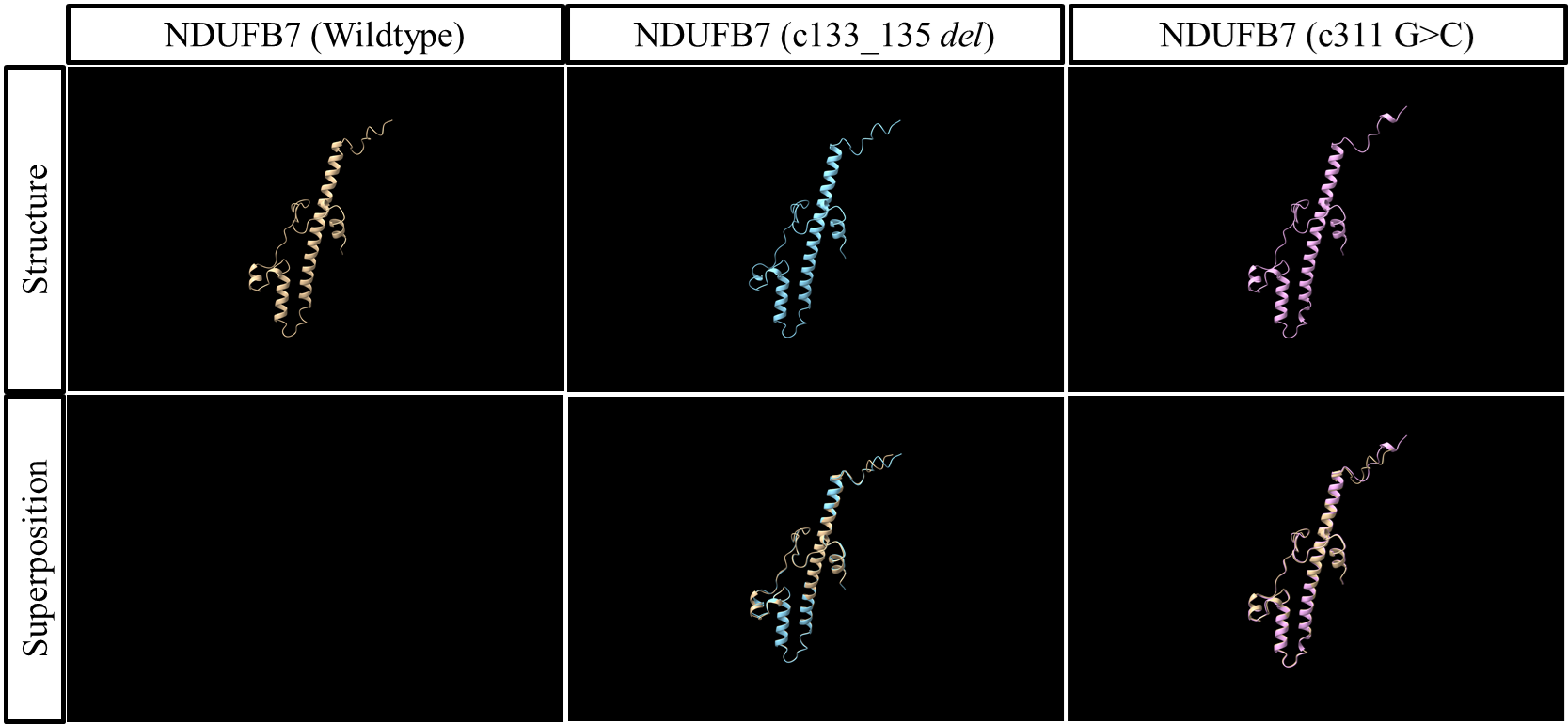


**Supplementary Figure 1. Structural prediction and alignment of NDUFB7 and its variants**
The three-dimensional structures of the NDUFB7 wild-type protein and its variants were predicted using AlphaFold. Structural alignments were conducted and visualized in ChimeraX to assess their similarity. The wild-type structure is depicted in yellow, while the c133_135 del and c311 G>C variant structures are shown in blue and pink, respectively. Superpositions with the wild-type structure are displayed below.
